# Identification of potential vaccines for use with microarray patches in low- and middle-income countries: An assessment from the Vaccine Innovation Prioritisation Strategy Alliance

**DOI:** 10.1016/j.vaccine.2025.126996

**Published:** 2025-05-10

**Authors:** Collrane Frivold, Birgitte Giersing, Jean-Pierre Amorij, Mercy Mvundura, Mateusz Hasso-Agopsowicz, Jessica Joyce Mistilis, Kristen Earle, Courtney Jarrahian, Marion Menozzi-Arnaud, Tiziana Scarna

**Affiliations:** a PATH, Seattle, Washington, USA; b World Health Organization, Immunization, Vaccines and Biologicals, Geneva, Switzerland; c United Nations Children's Fund, Supply Division, Vaccine Centre, Copenhagen, Denmark; d Gates Foundation, Seattle, Washington, USA; e Gavi, the Vaccine Alliance, Geneva, Switzerland

**Keywords:** Microarray patches, Microneedles, Vaccines, Prioritization, Coverage and equity, Low- and middle-income countries

## Abstract

**Introduction:**

Microarray patches (MAPs) have the potential to increase equitable vaccine coverage in low- and middle-income countries (LMICs). However, MAP developers and vaccine manufacturers have identified a barrier to development of MAPs for global health applications: the need for guidance on which vaccine MAPs would be of value to LMIC immunization programs. To address this gap, the Vaccine Innovation Prioritisation Strategy (VIPS) Alliance conducted a prioritization process to identify high-priority vaccines that could be delivered via MAPs in LMICs.

**Methods:**

We first compiled a reference list of vaccine targets through desk research, then filtered these targets based on route of administration, market distribution, existing interest from a global/regional health organization, whether the vaccine would address a specific global health priority or stakeholder agenda, development status, and potential MAP use cases. To further down-select the list, we consulted an external advisory group and evaluated the potential regulatory pathway, programmatic impact, and financial sustainability to define two priority levels.

**Results:**

From a reference list of 91 vaccine targets, we identified 21 with applicability to LMICs, which were further down-selected to a VIPS priority list of 11 vaccine targets grouped by priority level. Priority group 1 included vaccines against hepatitis B virus, measles-rubella/measles-mumps-rubella viruses, human papillomavirus, rabies virus, yellow fever, influenza virus (seasonal and pandemic), and SARS-CoV-2. Priority group 2 included vaccines against Group B streptococcus, *Neisseria meningitidis* A,C,W,Y,(X), *Salmonella Typhi*, and *Streptococcus pneumoniae*.

**Conclusions:**

These vaccine MAP priorities will inform the investment decisions of MAP developers, vaccine manufacturers, donors, and global health partners to better respond to country needs when designing their MAP portfolios. By providing a holistic assessment of the potential drivers for and key risks of developing specific vaccine MAPs, our findings have the potential to promote MAP development activities for vaccines that are priorities for LMICs.

## Highlights

• We identified vaccine applications for microarray patches to address LMIC needs.
• Starting from 91 vaccine targets, we identified 21 with applicability to LMICs.
• We defined priority groups by programmatic impact and financial sustainability.
• Results can inform technology developer/vaccine manufacturer investment decisions.
• Introducing these vaccine microarray patches could advance vaccine equity in LMICs.

## Graphical abstract

**Figure:**
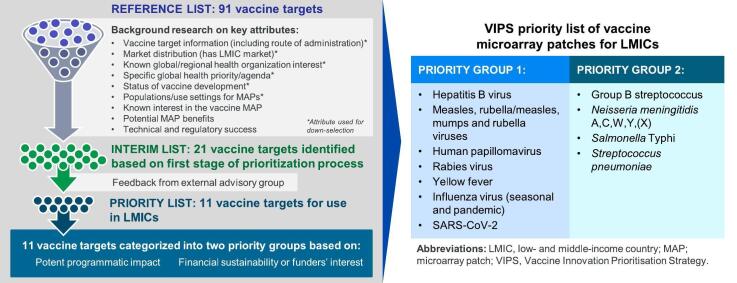
Graphical abstract summarizing a prioritization process conducted by the Vaccine Innovation Prioritisation Strategy to identify high-priority vaccines that could be delivered via microarray patches in low- and middle-income countries.

## 1 Introduction

Immunization programs play a crucial role in disease prevention and control, especially in low- and middle-income countries (LMICs). However, these programs face significant challenges due to inadequate suitability of vaccine product attributes for LMIC contexts, which can reduce their ability to meet coverage targets and achieve public health impact [1,2]. For instance, current vaccine presentations require cold chain storage and transport, but access to cold chain equipment and/or grid electricity to run the equipment can be limited in some areas. In addition, the number of injections being administered during a single vaccination visit is increasing, adding logistical complexity and reducing patient/caregiver acceptability. Moreover, vaccines are often stored in multidose containers, which leads to increased wastage or missed opportunities for vaccination when health workers are hesitant to open these containers for only the few individuals attending the vaccination session. The complexity of vaccine preparation and administration also varies by vaccine with some vaccines involving more complex steps, which can result in safety issues and increase the time it takes to administer vaccines.

In spite of efforts to strengthen country immunization programs and increase vaccine coverage globally, the proportion of children who receive three doses of a diphtheria-tetanus-pertussis–containing vaccine before their first birthday, a common metric for assessing immunization program strength, has stagnated at 84 % over the last decade, with declines in coverage after the onset of the COVID-19 pandemic (range: 81–86 %) [3]. In 2023, approximately 21 million children were un- or under-vaccinated, including 14.5 million zero-dose children (those who had not received any vaccines) [4,5]. Based on these trends, there is increasing recognition of the need for improved vaccine presentations, designed to meet the needs of LMIC immunization programs, and targeted solutions to expand vaccine access, reach remote and hard-to-reach populations, reduce missed opportunities for vaccination, and increase equitable vaccine coverage [5]. The COVID-19 pandemic has further highlighted the need for vaccine product innovations that improve ease of use; enable rapid, large-scale administration of vaccines during outbreak and/or pandemic response; and increase access to underserved populations, including hard-to-reach communities especially in conflict, vulnerable, or fragile settings.

Vaccine product innovations could help address some of the shortcomings of current vaccine presentations and ease delivery and/or administration to overcome vaccine coverage and equity shortfalls [2,6,7]. For instance, microarray patches (MAPs)—also referred to as microneedle patches or patches—consist of microscopic projections that deliver dry vaccine or drug when applied to the skin [[8], [9], [10]]. Compared to existing vaccine presentations, wherein the vaccine is stored in a multidose glass vial and administered with an autodisable needle and syringe, MAPs for vaccine applications offer several potential benefits, including increased thermostability, improved ease of use, reduced open-vial wastage, and improved safety (e.g., reduced administration errors, contamination, needle-stick injuries) [10]. By addressing barriers related to the product attributes of current injectable vaccines, MAPs could increase reach among un- and under-immunized populations and improve acceptability [[11], [12], [13], [14]]. Based on these potential programmatic benefits, MAPs have been prioritized by the Vaccine Innovation Prioritisation Strategy (VIPS) Alliance—composed of Gavi, the Vaccine Alliance (Gavi); the World Health Organization (WHO); the United Nations Children's Fund (UNICEF); the Gates Foundation; and PATH—as a transformative vaccine product innovation with the potential to increase vaccine coverage and equity in LMICs [15,16].

In 2021, the VIPS Alliance published a five-year action plan for vaccine MAPs (2021–2025) that describes key activities needed to accelerate the development and future uptake of vaccine MAPs for LMIC use [17]. Considering the significant financial investment that development of vaccine MAPs will require, activity 1 of the action plan is to “identify priority vaccines to be used with MAPs for LMICs.” This goal came as a response to industry consultations highlighting the need for clearer guidance to inform the business case for vaccine MAP investments, particularly for LMIC contexts. To address this gap, the VIPS Alliance conducted a prioritization process to identify high-priority vaccines for which there is likely a strong public health need for a MAP presentation in LMICs to help technology developers, vaccine manufacturers, and donors better respond to country needs.

## 2 Methods

**Fig. 1:**
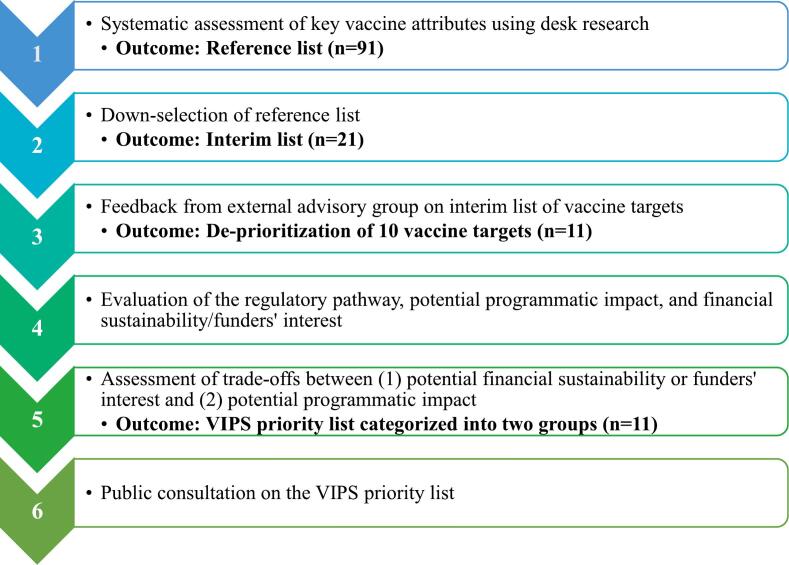
Overview of the microarray patch antigen prioritization process. Abbreviation: VIPS, Vaccine Innovation Prioritisation Strategy.

The prioritization process was conducted in 2021–2023. We used an explanatory sequential mixed methods approach whereby quantitative data were collected first, followed by collection of qualitative data [18,19]. An overview of the approach is shown in Fig. 1.

### 2.1 Reference list of vaccines

First, we compiled a comprehensive reference list of vaccine targets, defined as the name commonly used to refer to the vaccine or pathogen, including both licensed and investigational vaccine candidates.

The list was based on vaccine targets of interest to key stakeholders, including WHO, Gavi, UNICEF, the Pan American Health Organization, the Center for the Biomedical Advanced Research and Development Authority (BARDA), and the Coalition for Epidemic Preparedness Innovations (CEPI) (Supplemental Table 1). Vaccines that protect against different combinations of pathogens (e.g., measles only, measles-rubella [MR], measles-mumps-rubella [MMR]) were evaluated as unique vaccine targets. Additional vaccine targets were identified through review of publications on vaccine landscapes and pipeline products [[20], [21], [22]]. For many vaccines of interest, several different vaccine types (e.g., subunit, virus vector, live attenuated, nucleic acid) were identified, which we grouped together and evaluated under the same vaccine target to streamline the subsequent steps in the prioritization process (Supplemental Table 1).

### 2.2 Background research on potential vaccine targets for microarray patches

To inform the evaluation and prioritization of vaccine targets, we identified and compiled information on key vaccine attributes as shown in Table 1, which is organized into the following categories: (1) vaccine target information; (2) market distribution between LMICs and high-income countries (HICs); (3) known interest in the vaccine or disease from a global/regional health organization; (4) whether the MAP would address a specific global health priority or stakeholder agenda; (5) status of vaccine development; (6) populations and settings for potential use of vaccine MAPs; (7) known interest in the vaccine MAP expressed by a funder, MAP developer, and/or vaccine manufacturer; (8) potential benefits offered by the vaccine MAP; (9) attributes that could impact the probability of technical and regulatory success. These attributes were also selected based on the types of information that would be publicly available for all vaccine targets on the reference list. Using desk-based research, publicly available data on each vaccine target were collected through February 2022 and analyzed using Microsoft Excel. The Excel database was organized by vaccine target, with separate rows for each unique vaccine target/disease/vaccine type combination.

**Table 1:** Attributes included in background research on potential vaccine targets for microarray patches and rationale for their inclusion.

Attribute	Attribute definition	Rationale for including attribute
VACCINE TARGET INFORMATION		
Vaccine target or pathogen	Name commonly used to refer to the vaccine or pathogen.	
Disease	Name of the main disease(s) associated with the pathogen.	
Vaccine type	The different types of vaccines known to be in development for the target in question: Live attenuated virus/bacterium; viral vector (replicating/non-replicating); whole inactivated virus/bacterium (organism); subunit; virus-like particle; peptide; DNA; RNA; polysaccharide-protein conjugate vaccine. Information was collected from published literature or US National Library of Medicine ClinicalTrials.gov database [48].	Different vaccine types against the same target will be at different stages of clinical development. They will also have different properties, which could make them more or less suitable for use with MAPs.
Administration route	Route of administration: ID; IM; intranasal; intravenous; oral; subcutaneous. Information from the ClinicalTrials.gov database [48] or from other vaccine exemplars. If details could not be found, and other information suggested a likely route (e.g., use of aluminum salt adjuvants suggests that the vaccine is delivered intramuscularly, then “IM?” is entered. “N/A" (not applicable) was entered if no vaccines were identified.	Vaccines that are injected intradermally, intramuscularly, or subcutaneously are most likely to be compatible with MAPs.

MARKET DISTRIBUTION		
Market in LMICs	Qualitative assessment of whether the vaccine is used, or a vaccine MAP is likely to be used, in LMICs or HICs respectively, based on existing knowledge and a non-exhaustive literature search.	The primary purpose of the prioritization process is to select vaccine MAPs of relevance to LMICs. Vaccine MAPs that have an HIC market or both HIC and LMIC markets might be more commercially attractive to vaccine manufacturers and MAP developers.
Market in HICs		

KNOWN INTEREST IN VACCINE OR DISEASE FROM A GLOBAL/REGIONAL HEALTH ORGANIZATION		
WHO PDVAC priority	Vaccine targets regarded as priority by WHO's PDVAC [49].	Existing interest in the vaccine or pathogen from global/regional health organizations is used to indicate that there is a recognized need for a vaccine against the target in LMICs, and therefore potentially for a vaccine MAP, provided other criteria are met. Existing stakeholder interest might also increase the likelihood of funding and support for vaccine MAP development.
WHO PDR engaged	Vaccine targets with which the WHO PDR team is involved [50].	
WHO R&D Blueprint	Vaccine targets in the WHO R&D Blueprint portfolio [51].	
Gavi funded	Vaccines currently funded by Gavi [52].	
Gavi VIS	Vaccines under review by Gavi's VIS [53].	
UNICEF procured	Vaccines procured by UNICEF [54].	
PAHO procured	Vaccines procured by PAHO [55].	
CEPI portfolio	Pathogen targets with vaccines funded by CEPI. The pathogen target is captured, not just the individual vaccine targets that are funded by CEPI and are within the CEPI portfolio [56].	
CARB-X	Pathogen targets with vaccines funded by CARB-X. The pathogen target is captured, not just the specific candidates within the CARB-X portfolio [57].	
BARDA	Pathogen targets with vaccines funded by BARDA [58]. The pathogen target is captured, not just the specific candidates within the BARDA portfolio.	
Other funders	Pathogen targets with vaccines funded by other global health organizations (e.g., Wellcome Trust, Bill & Melinda Gates Foundation).	

SPECIFIC GLOBAL HEALTH PRIORITY/AGENDA		
Vaccine needed to combat antimicrobial resistance	Records vaccine targets needed to combat antimicrobial resistance from: • Wellcome Trust and Boston Consulting Group report [59]. Captures pathogens with high, medium, and low urgency of threat. • WHO list of priority pathogens for R&D of new antibiotics [60]. Captures pathogens with critical, high, or medium threat assessments. Pathogens listed as priority targets for vaccines due to risk of antimicrobial resistance [61].	The criteria represent specific global health needs that have already been identified and potentially could be applications for vaccine MAPs. This approach is used as an alternative to quantitative measures of burden of disease, which are not available for all pathogens being considered.
Elimination agenda	Records whether an elimination agenda has been announced for the pathogen by PAHO, WHO, or another global health body.	
Epidemic/pandemic potential	Records whether the pathogen has the potential to cause epidemics or pandemics.	

STATUS OF VACCINE DEVELOPMENT		
Prequalified	Vaccines against the target are WHO prequalified. From WHO list of prequalified vaccines [62].	Vaccines in different stages of development will be more or less appealing for use with vaccine MAPs depending on the perspective of the stakeholder (e.g., vaccines in phase 3 or later have demonstrated clinical proof of concept and so have a higher probability of success, reducing risk for vaccine manufacturers and MAP developers).
Licensed	Vaccines against the target have national regulatory authority approval, but are not prequalified.	
Phase 3	Vaccine candidates against the target are in phase 1, 2, or 3 clinical trials. Information from the ClinicalTrials.gov database [48]. Vaccines were included if clinical trials with the vaccine are listed as “active” and/or “completed,” and the information has been updated since January 2019. Older entries in the ClinicalTrials.gov database were not included.	
Phase 2		
Phase 1		
Preclinical	Vaccine candidates against the target are in preclinical development. For many vaccines, this information was not collected. Preclinical pipelines can be extensive and change rapidly, and it can be difficult to determine which candidates are being considered for clinical testing and which are research-level projects.	

POPULATIONS AND SETTINGS FOR POTENTIAL USE WITH VACCINE MAPS		
Outreach/campaign	A significant proportion of the vaccine's use is believed to be in outreach or campaign settings where the vaccination strategy involves reaching a targeted population with a single vaccine. Information obtained from WHO position papers [63], WHO preferred product characteristics [64], WHO target product profiles [64], and/or published literature.	Different vaccination settings or target populations might be more suitable for vaccine MAP use, or MAPs might offer more potential gains based on their attributes. All settings other than “RI usually at fixed post” were classified as “special strategies.”
Birth-dose	Records whether the vaccine is administered as a birth-dose.	
Routine immunization, usually at fixed health post	The vaccine is given at scheduled RI sessions that target multiple populations with multiple vaccines, usually at a fixed post or health care facility. WHO-recommended RI schedules were used [65], or national immunization schedules where available for HICs.	
Adolescents (or adults)	Is the vaccine primarily intended for immunization of adolescents and/or adults? If the vaccine can be given to adolescents/adults as part of an emergency campaign in all age groups, this is captured in outreach/campaign.	
Maternal	Records whether the vaccine has a specific indication for maternal immunization and recommendation for use during pregnancy.	
Older adults	Records whether the vaccine has a specific indication for use in older adults.	
Travel/migrants	Records whether the vaccine is recommended or required for travelers.	
Booster dose(s)	Records whether additional doses of the vaccine are recommended throughout life.	

KNOWN FUNDER/VACCINE MANUFACTURER/MAP DEVELOPER INTEREST IN VACCINE MAPS⁎		
Funders	Records any known, ongoing vaccine MAP projects being supported by funders. Based on nonconfidential information included in the VIPS Alliance MAP action plan and subsequent press releases or publications.	Captures existing interest expressed by stakeholders in vaccine MAPs (not simply vaccines).
Vaccine manufacturers	Records known vaccine MAP projects conducted by vaccine manufacturers or MAP developers, ongoing or completed, that are aimed at taking a candidate to phase 1 or later clinical trials. Based on nonconfidential information included in the VIPS Alliance MAP action plan and subsequent press releases or publications.	
MAP developers		

POTENTIAL BENEFITS OFFERED BY A VACCINE MAP⁎		
Dose-sparing/immunogenicity	Would the vaccine benefit from dose-sparing or improved immunogenicity (e.g., faster kinetics of immune response) that might result from administration by a vaccine MAP?	The potential benefits listed are attributes that might be associated with vaccine MAPs (depending on the vaccine and MAP format). They can be used to build a rationale that supports (or not) the likely value of the vaccine MAP in LMICs. The information captured can vary between different vaccines and vaccine types against the same target, so could lead to selection of specific vaccine types or individual candidates for use with a MAP.
Thermostability	Would the vaccine benefit from improved thermostability that might result from use as a vaccine MAP?	
Missed opportunities	Is a vaccine MAP likely to result in fewer missed opportunities for vaccination?	
Ease of use/self-administration†	Would the vaccine benefit from improved ease of use or self-administration that might result from use as a vaccine MAP [66]?	
Acceptability	Is use of a vaccine MAP likely to improve acceptability of the vaccine?	
Avoid reconstitution	Would use of a vaccine MAP avoid reconstitution?	
Representative of vaccine type	Is the vaccine an exemplar of its type so that experience of formulating and using it with MAPs can be applied to other, similar vaccines or vaccines that use the same platform?	

ATTRIBUTES THAT COULD IMPACT THE PROBABILITY OF TECHNICAL AND REGULATORY SUCCESS⁎		
Target antigen known	Is the vaccine or target antigen(s) required for protection and to be delivered by the MAP already known?	Development will be much simpler and lower risk if the target is known and/or a vaccine candidate already exists.
Suitable candidate	Is there a suitable vaccine candidate that could be used for vaccine MAP development?	
Adjuvant free	Does the current formulation of the vaccine require an adjuvant?	Formulation for use with a MAP will be more straightforward and reactogenicity is likely to be less if there is not an adjuvant.
Mono−/low valency	Is the vaccine monovalent or of low valency (i.e., ≤ 4 valent)? Four-valent was selected as a threshold because several MAP developers are known to be developing seasonal quadrivalent influenza vaccines (4-valent), suggesting this is technically feasible for several MAP platforms.	Lower-valency vaccines are likely to be easier to develop.
Correlates of protection/immunogenicity endpoints	Are immunological correlates of protection known, or is it likely that phase 3 trials can have an immunogenicity endpoint (e.g., non-inferiority of antibody response)?	Correlates of protection and immunological endpoints will simplify clinical development compared to using an efficacy endpoint.
Positive clinical data	Are there positive data from clinical trials with the vaccine?	Positive clinical data, clinical experience with ID delivery, and previous clinical or preclinical work with a vaccine MAP will contribute to reducing the risk of developing a vaccine MAP.
Clinical ID delivery data	Has the vaccine been delivered intradermally, or is it being delivered this way in practice?	
MAP preclinical data	Have preclinical studies been conducted with MAPs and the vaccine in question?	
MAP clinical data	Have any clinical studies been conducted with MAPs and the vaccine in question?	

Abbreviations: BARDA, Center for the Biomedical Advanced Research and Development Authority; CARB-X, Combating Antibiotic-Resistant Bacteria; CEPI, Coalition for Epidemic Preparedness and Innovations; Gavi, Gavi, the Vaccine Alliance; HIC, high-income country; ID, intradermal; IM, intramuscular; LMICs, low- and middle-income countries; MAP, microarray patch; PAHO, Pan American Health Organization; PDR, Product and Delivery Research (WHO); PDVAC, Product Development Vaccine Advisory Committee (WHO); R&D, research and development; RI, routine immunization; UNICEF, United Nations Children's Fund; VIPS, Vaccine Innovation Prioritisation Strategy; VIS, Vaccine Investment Strategy (Gavi); WHO, World Health Organization.

⁎ Attributes in this category were not used as selection criteria to generate the interim list. See Table 2 for details on the selection criteria used to down-select from the reference list to the interim list.

† Using criteria and definitions applied in VIPS phase 2.

### 2.3 Down-selection of vaccine targets on the reference list to generate an interim list

To down-select from the initial reference list, we applied a series of filters to the background information collected on each vaccine target, as shown in Table 2. We first selected vaccines that are administered by intramuscular, intradermal, or subcutaneous injection, as vaccines administered orally, intranasally, or intravenously were assumed to be less suitable for MAP administration. Oral/intranasal formulations are also already needle-free and therefore would benefit less from a MAP presentation. We also filtered for vaccines that have or are expected to have an LMIC market, regardless of whether they have a market in HICs, as our focus was on vaccine MAPs that could be of value to LMIC markets. After that, we selected vaccines in which there is interest in the vaccine or disease area from a global/regional health organization (e.g., vaccines regarded as a priority by the WHO Product Development Vaccine Advisory Committee [PDVAC] for non-epidemic pathogens or WHO R&D Blueprint for outbreak pathogens). Moreover, we selected vaccine targets that address an already established global health need (e.g., to combat antimicrobial resistance [23]) as a proxy for general global health relevance of the vaccine target. Next, we selected licensed vaccines or candidates in phase 3 clinical development to increase the likelihood of MAP technical and regulatory success compared to using an earlier-stage antigen for which safety/immunogenicity data have not yet been generated with another route of administration (e.g., intramuscular, intradermal, or subcutaneous). For outbreak pathogens, we included candidates in phase 2 if they were considered a high priority according to the WHO R&D Blueprint [24] or CEPI [25]. Finally, we selected vaccines that are often administered outside of routine, fixed-post settings for infants since MAPs have the greatest potential for programmatic benefit when administered to other target populations (e.g., birth-dose, adolescents, adults, pregnancy) and outside facilities (e.g., outreach, campaigns, community settings). This filtering process resulted in an interim list of vaccine targets for MAP delivery that were evaluated in a second phase of the prioritization process.

**Table 2:** Selection criteria for evaluation of the reference list (*n* = 91) of potential vaccines for use with microarray patches and down-selection to the interim list (*n* = 21).

Category of attributes	Selection criteria	Rationale
Vaccine target information	Selected vaccines that are administered by IM, ID, or SC injection.	Vaccines that are delivered orally, intranasally, or intravenously are not likely to be suitable for MAP administration compared to vaccines currently administered by IM, ID, or SC injection. Current vaccines administered via the oral and intranasal routes are typically live attenuated organisms that deliver vaccine directly to the mucosal surfaces that are the site of transmission (e.g., respiratory tract, intestine). The vaccine antigen then replicates in the mucosal epithelium to induce local mucosal immunity. Although live attenuated vaccines can also be administered via the IM, ID, or SC route, these routes typically induce only a systemic immune response instead of both systemic and mucosal immunity. Vaccines administered intranasally or orally would likely also benefit less from a MAP presentation since these formulations are already needle-free. Intravenous administration of vaccines is rare and typically only considered if other routes are not technically feasible, which could further reduce the probability of technical success when reformulating an intravenous vaccine into a MAP presentation.
Market distribution	Selected LMIC market.	Captures vaccines that have or are expected to have a market in LMICs, whether or not they have a market in high-income countries.
Global/regional health organizations with known interest in vaccine or disease	Selected vaccines for which there is interest or funding from any of the stakeholders listed.	Existing stakeholder interest in the vaccine or pathogen indicates knowledge of the target and that there is need for a vaccine, and therefore potentially a need for a vaccine MAP, provided other criteria are met. Existing stakeholder interest might also increase likelihood of funding and support for vaccine MAP development.
Specific global health priority	Selected vaccines that meet any of the criteria.	There is already an identified specific global health need for these vaccines.
Status of vaccine development	Selected vaccines that are in phase 3 or later (including licensed vaccines). *Phase 2 or later for outbreak pathogens.*	This selects for vaccines that are either WHO prequalified, licensed, or have successfully completed phase 2 trials. They should therefore have demonstrated some level of efficacy or proof of concept in the clinic. This reduces some of the risks associated with developing a vaccine MAP. Vaccines with outbreak potential were not excluded if they were in phase 2, since these pathogens are considered high priority in the WHO R&D Blueprint or by the Coalition for Epidemic Preparedness Innovations, with a mandate on outbreak diseases.
Populations and settings for potential use of vaccine MAPs	Selected vaccines that meet any of the subheading criteria, except routine immunization for children.	MAPs could be used in any vaccination setting or population. The potential benefits of MAPs are likely to be greater in settings other than routine immunization of infants in fixed-post settings (i.e., special strategies). Therefore, there might be a stronger rationale for developing MAPs with these vaccines.

Abbreviations: ID, intradermal; IM, intramuscular; LMICs, low- and middle-income countries; MAP, microarray patch; R&D, research and development; SC, subcutaneous; WHO, World Health Organization.

### 2.4 In-depth analysis of the interim list to generate the Vaccine Innovation Prioritisation Strategy priority list

The business case for developing vaccine MAPs specifically for LMIC markets is complex and requires trade-offs between potential programmatic benefits, technical and regulatory complexity, and commercial viability. To capture these, we further refined the interim list of vaccine targets through a virtual group consultation with an external advisory group (*n* = 13) with expertise in vaccine policy, program implementation, supply chain, vaccine financing and commercialization, and product development, including current/former members of WHO's Strategic Advisory Group of Experts on Immunization and PDVAC (Supplemental Table 2).

After de-prioritizing vaccines based on expert feedback, we qualitatively mapped the remaining vaccine targets along three dimensions to generate a final priority list: (1) estimated complexity of the regulatory pathway based on identified surrogates of efficacy, correlates of protection, and immunological endpoints; (2) potential programmatic impact based on the ability of the MAP to address current challenges faced by LMIC immunization programs, which vaccine programs country stakeholders perceived would benefit the most from MAPs, and the number of potential programmatic benefits offered by a MAP; and (3) financial sustainability or funders' interest based on the potential for a dual market where a MAP product is intended for both LMIC and HIC markets, known interest in the vaccine MAP (from a funder, MAP developer, and/or vaccine manufacturer), and vaccine MAP stage of development. Using the information collected on each vaccine target and from the previously conducted VIPS country consultations [26], team members with knowledge of these issues assessed the considerations described above and qualitatively ranked them from “low” to “high” for each remaining vaccine target (Supplemental Tables 3–5). The initial rankings were assigned collaboratively by two individuals and then finalized based on group consensus after review and discussion with the broader VIPS team. For the estimated complexity of the regulatory pathway, a “low” score suggested that MAP development would be more favorable. For the potential programmatic impact and the financial sustainability or interest from potential funders, a “high” score suggested that MAP development would be more favorable. For each vaccine target on the interim list, we also generated summaries describing the potential drivers for and key risks of developing a vaccine MAP presentation.

VIPS launched a public consultation in January 2023 to provide an opportunity for a wider range of stakeholders—including vaccine manufacturers, technology developers, and global health stakeholders—to share their feedback on the final VIPS priority list. This process included disseminating the outcomes of the prioritization process and requesting feedback online through TechNet-21, a global network of immunization professionals [27].

## 3 Results

### 3.1 Down-selection from the reference list to the interim list

**Fig. 2:**
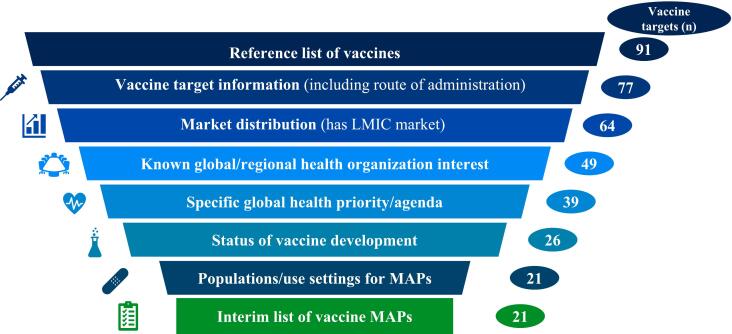
Outcomes of the reference list evaluation (*n* = 91) used to generate the interim list of vaccine targets for use with microarray patches (*n* = 21). Abbreviations: LMIC, low- and middle-income country; MAP, microarray patch.

Fig. 2 summarizes the outcomes of the prioritization process from the starting reference list (*n* = 91) to the interim list of vaccines for use with MAPs (*n* = 21), including the number of vaccine targets retained after each filtering step. A summary of the data collected on each selection criterion used to generate the interim list of vaccine targets is shown in Table 3.

**Table 3:** Summary of vaccine targets included on the interim list for use with microarray patches (n = 21).

Vaccine target	Vaccine type	Route of administration	Status of development for most advanced candidate	Global/regional health organization interest	Specific global health priority	Special strategies for use (outside RI for children)⁎	MAP development activities (as of February 2022)	Inclusion in VIPS priority list (yes/no)
Chikungunya virus	Subunit (VLP)	IM	Phase 3	CEPI	Epidemic potential	Campaign/outreach; adolescent and/or adult immunization [67,68]	MAP preclinical data available.	No
	LAV	IM	Phase 3					
	WIV	IM	Phase 3					
	Virus vector (replicating)	IM	Phase 3					
Ebola virus	Virus vector (replicating)	IM	PQ	WHO R&D Blueprint; BARDA	Epidemic potential	Campaign/outreach [69]	MAP preclinical data available.	No
	Virus vector (non-replicating)	IM	PQ					
Group B streptococcus	PS-PCV	IM	Phase 3 (planned)	WHO PDVAC	AMR	Maternal immunization	No GBS MAPs are known to be in development.	Yes
Hepatitis B virus	Subunit	IM	PQ	Gavi VIS; UNICEF; PAHO	Elimination agenda	Birth-dose; adolescents; adults (HICs)	MAP preclinical and clinical data available. Two phase 1 studies with a hepatitis B vaccine MAP were conducted in adults [70].	Yes
Human papillomavirus	Subunit (VLP)	IM	PQ	Gavi; UNICEF; PAHO	Elimination agenda	Campaign/outreach; RI in adolescents	MAP preclinical data available. HPV MAP TPP available [71].	Yes
Influenza virus (pandemic)	Subunit/split	IM	PQ	BARDA (Gavi briefing)	Pandemic potential	Campaign/outreach; all age groups including adults and maternal	Pandemic influenza MAP preclinical work planned. Seasonal influenza vaccine MAPs have been evaluated in multiple phase 1 clinical studies [72,^73^,74].	Yes
Malaria (*Plasmodium falciparum*)	Subunit	IM	Licensed	WHO PDVAC	Elimination agenda	Campaign/outreach; RI in adolescents and/or adults; maternal immunization potential [75]	MAP preclinical data available.	No
Measles-containing vaccines *MR and MMR considered unique vaccine targets (n = 2)*	LAV (measles-rubella)	SC	PQ	Gavi; UNICEF	Elimination agenda; epidemic potential	Campaign/outreach	MAP preclinical data available. MR MAP TPP available [76]. Since February 2022, the results of phase 1 and 1/2 MR MAP studies have been published [77,78]. Additional phase 2 studies are planned.	Yes
	LAV (MMR)	SC	PQ	Gavi; UNICEF; PAHO	Elimination agenda; epidemic potential	Campaign/outreach		
MERS-CoV	Nucleic acid	IM	Phase 2	WHO R&D Blueprint; CEPI; BARDA	Epidemic/ pandemic potential	Campaign/outreach	No MERS-CoV MAPs are known to be in development.	No
*Mycobacterium tuberculosis*	LAB (next-generation)	ID	Phase 3	Gavi; WHO PDVAC	AMR; elimination agenda; epidemic potential	Birth-dose; adolescents and/or adults; travel	MAP preclinical data available.	No
	Subunit	IM	Phase 3					
	LAB (BCG)	ID	PQ					
*Neisseria meningitidis* A,C,W,Y(X)	PS-PCV	IM	PQ (A,C,W,Y) Phase 3 (A,C,W,Y,X)	Gavi VIS; PAHO	Elimination agenda; epidemic potential	Campaign/outreach, RI in adolescents (HICs); travel; booster doses	No meningococcal MAPs are known to be in development.	Yes
Poliovirus	WIV (IPV)	IM; ID	PQ	Gavi; GAVI VIS; UNICEF; PAHO	Elimination agenda; epidemic potential	Campaign/outreach	MAP preclinical data available.	No
Rabies virus	WIV	IM; ID	PQ	WHO PDR; Gavi VIS; UNICEF; PAHO	Elimination agenda	Campaign/outreach; adolescents and adults; travel	MAP preclinical data available. Rabies MAP TPP available [79].	Yes
Rift Valley fever virus	WIV	IM	Phase 2	WHO R&D Blueprint; CEPI; BARDA	Epidemic/ pandemic potential	Campaign/outreach	No Rift Valley fever MAPs are known to be in development.	No
	LAV	IM	Phase 2					
SARS-CoV-2	DNA	ID	Licensed	WHO R&D Blueprint; CEPI, BARDA	Pandemic potential	Campaign/outreach; travel; booster doses	MAP preclinical data available. Since February 2022, phase 1 and 2a studies have been initiated or completed [80]. COVID-19 MAP TPP available [81].	Yes
	WIV	IM	Licensed					
	Subunit	IM	Licensed					
	RNA	IM	Licensed					
	Virus vector (non-replicating)	IM	Licensed					
	Subunit (VLP)	IM	Phase 3					
	Virus vector (replicating)	IM	Phase 2					
*Salmonella Typhi*	PS; PS-PCV	IM	PQ	Gavi; UNICEF; CARB-X; other	AMR; epidemic potential	Campaign/outreach; travel	As of February 2022, TCV MAPs were not in development, but preclinical studies have been initiated and future clinical studies planned. The VIPS Alliance conducted a TCV MAP FVVA as part of the action plan for vaccine MAPs.	Yes
*Streptococcus pneumoniae*	PS-PCV	IM	PQ	Gavi; UNICEF; PAHO	AMR	Older adults (HICs)	MAP preclinical data available.	Yes
Tetanus-diphtheria (low-dose diphtheria)	Subunit	IM	PQ	Gavi; UNICEF; PAHO	Elimination agenda	Adolescent; adult; maternal; booster dose(s)	MAP preclinical data available.	No
Yellow fever	LAV	IM; SC	PQ	Gavi; UNICEF; PAHO	Epidemic potential	Campaign/outreach; travel	No yellow fever MAPs are known to be in development.	Yes
Zika virus	DNA	ID	Phase 2	WHO R&D Blueprint; BARDA	Epidemic/ pandemic potential	Campaign/outreach	No Zika MAPs are known to be in development for DNA or mRNA vaccines. MAP preclinical data are available for a whole inactivated Zika vaccine.	No
	mRNA	IM	Phase 2					

Abbreviations: AMR, antimicrobial resistance; BARDA, Center for the Biomedical Advanced Research and Development Authority; BCG, bacillus Calmette-Guérin; CARB-X, Combating Antibiotic-Resistant Bacteria; CEPI, Coalition for Epidemic Preparedness and Innovations; COVID-19, coronavirus disease 2019; FVVA, full value of vaccine assessment; Gavi, Gavi, the Vaccine Alliance; GBS, Group B streptococcus; HIC, high-income country; HPV, human papillomavirus; ID, intradermal; IM, intramuscular; IPV, inactivated poliovirus vaccine; LAB, live attenuated bacterium; LAV, live attenuated virus; MAP, microarray patch; MERS-CoV, Middle East respiratory syndrome coronavirus; MMR, measles-mumps-rubella; MR, measles-rubella; PAHO, Pan American Health Organization; PDR, Product Development and Research (WHO); PDVAC, Product Development Vaccine Advisory Committee (WHO); PQ, prequalified; PS-PCV, polysaccharide-protein conjugate vaccine; R&D, research and development; RI, routine immunization; SARS-CoV-2, severe acute respiratory syndrome coronavirus 2; SC, subcutaneous; TCV, typhoid conjugate vaccine; TPP, target product profile; UNICEF, United Nations Children's Fund; VIPS, Vaccine Innovation Prioritisation Strategy; VIS, Vaccine Investment Strategy (Gavi); VLP, virus-like particle; WHO, World Health Organization; WIV, whole inactivated virus.

⁎ Only “special strategies are listed.” RI for children was not a selection criterion so is not shown, even if the vaccine is used for this purpose.

### 3.2 Down-selection from the interim list to the final Vaccine Innovation Prioritisation Strategy priority list

Based on feedback from the external advisory group, we narrowed down the interim list of 21 vaccine targets to 11 priority vaccines for MAPs. At this stage, we also collapsed measles-containing vaccines (MCVs) into a single vaccine target (e.g., MR or MMR). Ten vaccines (*n* = 10/21) were excluded based on low commercial viability due to the expected price premium a MAP presentation would require compared to the current presentation, small market size, or the lack of a defined surrogate of efficacy, which could impact the likelihood of technical and regulatory success. See Table 4 for details.

**Table 4:** Rationale for de-prioritizing vaccine targets on the interim list based on feedback from the external advisory group (n = 10).

Vaccine category	Vaccine target(s)	Rationale for de-prioritization
Legacy *High volumes of vaccines at low unit prices available*	*Mycobacterium tuberculosis* (BCG)	The low price point of BCG makes it an unfavorable target for MAPs due to the expected price premium that would be required for a MAP presentation compared to the current presentation.
	Poliovirus (IPV)	In the next ten years, it is likely that there will not be a large market for IPV as a stand-alone product as it may be replaced by the hexavalent vaccine.
	Tetanus-diphtheria (Td, low-dose diphtheria toxoid)	The low price point of Td makes it an unfavorable target for MAPs due to the expected price premium that would be required for a MAP presentation compared to the current presentation. Due to the low cost of the antigen, a Td MAP may face similar challenges to the TT CPAD regarding uptake, which was introduced in LMICs for maternal immunization targeting hard-to-reach populations, but later discontinued with the low antigen cost being a key barrier to uptake.⁎
Evolving *Not commoditized/higher-price vaccines, or vaccines still in development*	Malaria	There is no surrogate of efficacy identified for these vaccines, so both would be very risky choices from a MAP development perspective.
	*Mycobacterium tuberculosis* (next-generation)	
Outbreak response *Vaccine targets with unpredictable demand driven by outbreaks*	Chikungunya virus	All outbreak vaccines present a very challenging business case, and some are still at a relatively early stage of development. In addition, clinical trials are complex for these vaccine targets, since capturing enough cases/disease transmission to assess rare outcomes in a clinical trial can be challenging. Therefore, only influenza (pandemic and seasonal) and SARS-CoV-2 were included for further evaluation, as representative antigens of outbreak vaccines because they are also used in endemic settings.
	Ebola virus	
	MERS-CoV	
	Rift Valley fever virus	
	Zika virus	

Abbreviations: BCG, bacillus Calmette-Guérin; IPV, inactivated poliovirus vaccine; LMIC, low- and middle-income country; MAP, microarray patch; MERS-CoV, Middle East respiratory syndrome coronavirus; SARS-CoV-2, severe acute respiratory syndrome coronavirus 2; Td, tetanus-diphtheria with low-dose diphtheria toxoid; Tdap, tetanus-diphtheria-acellular pertussis; TT, tetanus toxoid.

⁎ Although most LMICs use Td, there could be interest in a Tdap MAP in the future, which also protects against pertussis, if countries switch from Td to Tdap and it becomes available through Gavi. However, at present, Tdap demand is driven by high-income and upper-middle-income countries. In these markets, the Tdap price per dose ranged from $12.72–$21.54 in 2019–2021, which could increase a stakeholders' willingness to pay a price premium compared to Td, which is a relatively low-priced vaccine with a weighted average price of $0.10 per dose through UNICEF and PAHO in 2021. Although a Tdap MAP has dual-market potential, the additional pertussis antigen would also increase the technical complexity of developing a tetanus-toxoid-containing vaccine MAP.

**Fig. 3:**
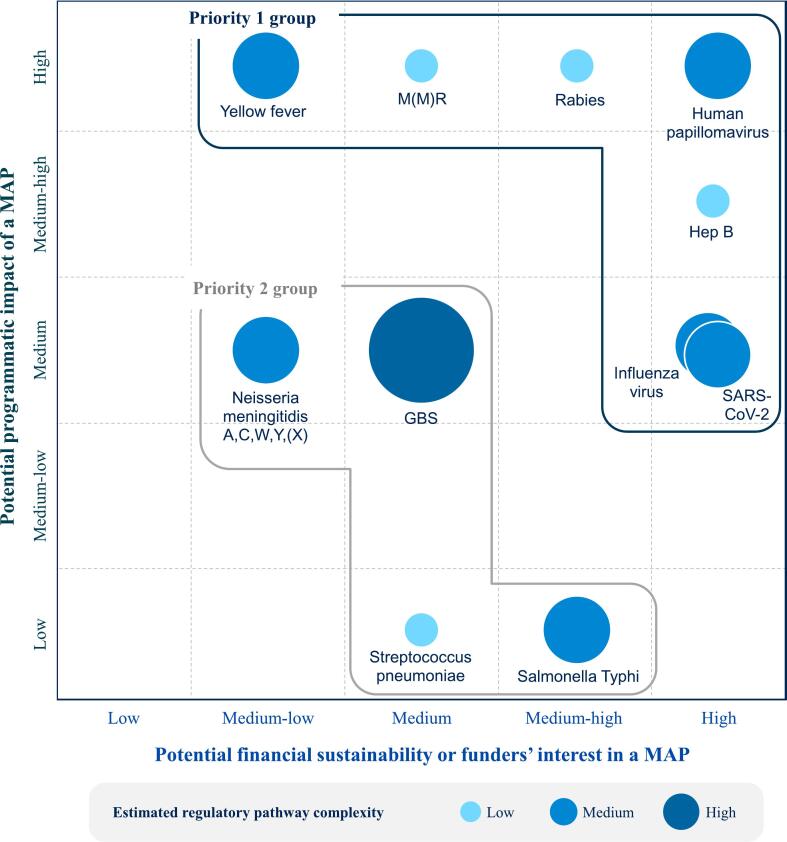
Categorization of priority groups for vaccine microarray patches based on trade-offs between the (1) potential financial sustainability or funders' interest and (2) potential programmatic impact. The potential financial sustainability or funders' interest is displayed on the x-axis; the potential programmatic impact is displayed on the y-axis. Vaccine targets in the top righthand corner are the highest priority based on these considerations. Priority group 1 is outlined in dark blue and priority group 2 is outlined in gray. The fill color and size of the circles represents the estimated complexity of the regulatory pathway (low; medium; high). The overlapping circles for influenza virus and SARS-CoV-2 represent potential synergies between outbreak pathogens. See Table 5 and Supplemental Tables 3–5 for further details on the qualitative rankings presented within this figure. Abbreviations: GBS, Group B streptococcus; Hep B, hepatitis B; MAP, microarray patch; M(*M*)R, measles-mumps-rubella; SARS-CoV-2, severe acute respiratory syndrome coronavirus 2. (For interpretation of the references to color in this figure legend, the reader is referred to the web version of this article.)

The trade-offs between the anticipated complexity of the regulatory pathway, the potential programmatic impact, and the financial sustainability or interest of potential funders for the 11 remaining vaccine targets are shown in Table 5. For instance, while the estimated regulatory pathway complexity of a *Streptococcus pneumoniae* MAP was considered “low”, the potential programmatic impact was also “low”, and the potential financial sustainability or funders' interest was “medium”. As such, while the MAP development pathway may be more straightforward for an *S. pneumoniae* MAP than other MAP products, the added public health value was also considered lower since a MAP presentation is expected to offer fewer potential benefits for *S. pneumoniae* compared to other vaccine targets. In contrast, for human papillomavirus (HPV) vaccine MAPs, while the estimated complexity of the regulatory pathway was considered more complex (“medium”), the potential programmatic impact and potential financial sustainability or funders' interest were higher. Based on these qualitative considerations (Table 5; Supplemental Tables 3–5), we identified two priority levels, defined as priority groups 1 and 2, based on potential programmatic impact of the vaccine MAP versus the feasibility of introducing the MAP in LMIC markets based on potential financial sustainability or funders' interest (Fig. 3). Priority group 1 included vaccine targets with “high” potential programmatic impact and/or “high” potential financial sustainability or interest from funders. All other vaccine targets that did not meet this definition were included in priority group 2. The feedback received through the public consultation aligned with the outcomes of the prioritization process and no modifications were made to the VIPS priority list based on the public consultation**.**

**Table 5:** Additional considerations on regulatory pathway, potential programmatic impact, and financial sustainability/funders' interest used to generate the Vaccine Innovation Prioritisation Strategy priority list (*n* = 11).⁎

Potential vaccine targets for use with MAPs		Estimated regulatory pathway complexity	Potential programmatic impact	Potential financial sustainability or funders' interest
Legacy vaccines: High volumes of vaccines available at a low unit price				
Hepatitis B virus		Low	Medium-high	High
Measles and rubella/measles, mumps, and rubella viruses	Measles and rubella	Low	High	Medium
	Measles, mumps, and rubella	Medium	High	High
Rabies virus		Low	High	Medium-high
*Salmonella Typhi*		Medium	Low	Medium-high
Yellow fever		Medium	High	Medium-low

Evolving vaccines: Not commoditized/higher-priced vaccines, or vaccines still in development				
Group B streptococcus (*Streptococcus agalactiae*)		High	Medium	Medium
Human papillomavirus		Medium	High	High
*Neisseria meningitidis* A,C,W,Y,(X)	*Neisseria meningitidis* A,C,W,Y	Medium	Medium	Medium-low
	*Neisseria meningitidis* A,C,W,Y,X	Medium	Medium	Medium-low
*Streptococcus pneumoniae*		Low	Low	Medium

Outbreak vaccines: Vaccine targets with unpredictable demand driven by outbreaks				
Influenza virus (pandemic and seasonal)		Medium	Medium	High
SARS-CoV-2		Medium	Medium	High

Abbreviations: MAP, microarray patch; SARS-CoV-2, severe acute respiratory syndrome coronavirus 2.

⁎ Estimated regulatory pathway complexity defined as low: surrogate of efficacy identified; medium: correlate of protection or immunological endpoint identified; high: no correlate of protection nor immunological endpoint identified. Potential programmatic impact evaluated based on the number of top five immunization challenges addressed by MAPs; ranking of whether the immunization program could benefit from a MAP presentation; and number of potential benefits offered by MAPs. Potential financial sustainability or funders' interest based on the potential for a dual market; known interest in the vaccine MAP from a funder, MAP developer, and/or vaccine manufacturer; vaccine MAP stage of development. See Supplemental Tables 3 through 5 for details on how these considerations were scored.

### 3.3 Overview of vaccine targets on the Vaccine Innovation Prioritisation Strategy priority list

The final VIPS priority list of vaccine targets for MAPs is shown in Table 6. Priority group 1 included seven vaccines against the following pathogens: hepatitis B virus, MR/MMR viruses, HPV, rabies virus, yellow fever, influenza virus (seasonal and pandemic), and SARS-CoV-2. Priority group 2 included four vaccines against the following pathogens: Group B streptococcus (GBS), *Neisseria meningitidis* A,C,W,Y,(X), *Salmonella Typhi*, and *S. pneumoniae*. Potential drivers for developing MAPs for the vaccines on the priority list, including the current unmet need and potential MAP benefits, are summarized below (Table 3; Supplemental Tables 5–6). See the Appendix for further details on potential drivers for and key risks of developing these vaccine MAP applications.

**Table 6:** Proposed Vaccine Innovation Prioritisation Strategy priority list of vaccine targets for microarray patches by priority group.

Priority group	Vaccine target
Priority group 1	Hepatitis B virus
	Measles and rubella/measles, mumps, and rubella viruses
	Human papillomavirus
	Rabies virus
	Yellow fever
	Influenza virus (seasonal and pandemic)
	SARS-CoV-2
Priority group 2	Group B streptococcus (*Streptococcus agalactiae*)
	*Neisseria meningitidis* A,C,W,Y,(X)
	*Salmonella Typhi*
	*Streptococcus pneumoniae*

Abbreviation: SARS-CoV-2, severe acute respiratory syndrome coronavirus 2.

### 3.4 Hepatitis B virus

Administering a birth-dose of the hepatitis B vaccine can prevent perinatal infection in infants. Globally, hepatitis B birth-dose coverage remains low; 45 % of infants were protected in 2023 [28]. Coverage rates were lowest among countries with a high proportion of out-of-facility births. As of 2023, 80 LMICs had introduced universal hepatitis B birth-dose vaccination [28]. MAPs have the potential to increase timeliness of hepatitis B birth-dose vaccination (within 24 hours of delivery) to improve vaccine effectiveness. Moreover, due to increased ease of use, MAPs could expand access by facilitating home-based delivery by midwives or birth attendants, where policy would allow, to ensure more infants are protected against perinatal infection. In addition to the birth-dose application, monovalent hepatitis B vaccines are recommended for at-risk adolescents and adults, including in HICs, which could expand the potential market size of hepatitis B MAPs.

### 3.5 Measles, rubella/measles, mumps, and rubella viruses

High coverage rates for MCVs (at least 95 %) are required to eliminate measles transmission [*29*]. Since 2010, coverage of the first dose of MCV (MCV1) has stalled at approximately 85 % (range: 84–86 %) [30]. Global coverage decreased further after the onset of the COVID-19 pandemic, from 86 % in 2019 to 81 % in 2020, and has not yet fully recovered, with 83 % MCV1 coverage reported in 2023 [4,31]. In addition to these reductions in global MCV1 coverage, inequities in vaccine coverage persist, with the greatest burden of morbidity and mortality among children in the lowest-income and most difficult-to-reach communities. A MAP presentation could offer several potential benefits, including increased thermostability, improved ease of use, increased acceptability, reduced open-vial wastage, and improved safety, which could increase equitable vaccine coverage, enable delivery by lesser-trained personnel in community settings, and facilitate reaching measles elimination goals [32,33]. Although MR is used in LMICs, most upper-middle- and high-income countries use costlier MMR or measles-mumps-rubella-varicella combination vaccines in their immunization programs. As such, developing an MMR MAP could increase commercial viability due to the higher price per dose of the lyophilized MMR vaccine and by providing access to markets in upper-middle- and high-income countries [32,34]. However, increasing the valency of a vaccine MAP also reduces the probability of technical success, since each antigen requires additional formulation and stability work.

### 3.6 Human papillomavirus

In 2020, WHO Member States adopted a global strategy toward global cervical cancer elimination, committing to introduce the HPV vaccine in all countries and reach a target of 90 % global vaccine coverage by 2030 [35]. As of 2023, 143 Member States had introduced the vaccine [28]. However, only 27 % of girls globally had received at least one HPV vaccine because coverage is suboptimal in many countries where the vaccine has been introduced and introduction has not yet taken place in >50 countries [28]. The cervical cancer burden is greatest in LMICs, and individuals at greatest risk of cervical cancer are the least likely to have access to the vaccine. HPV MAPs have the potential to facilitate vaccine delivery to adolescents in schools and administration settings outside of facilities, which could enable greater coverage. MAP delivery may also improve acceptability among adolescents for whom a needle-free presentation may be highly relevant given the higher prevalence of needle-phobia and post-injection syncope. Furthermore, the HPV vaccine market in HICs could facilitate a dual market and increase the commercial viability of HPV MAPs.

### 3.7 Rabies virus

Rabies causes an estimated 59,000 deaths annually [36]. Every year, more than 15 million people, mostly children, receive post-exposure prophylaxis (PEP) following dog bites, but many do not complete the full vaccination series [37,38]. A rabies MAP could simplify PEP administration and ensure completion of the vaccination series by enabling administration in a community setting and removing the need to return to a health facility for each dose. While the greatest unmet need in LMICs is for PEP, a MAP presentation could also expand access to pre-exposure prophylaxis (PrEP). Increased PrEP availability would provide additional programmatic benefits by eliminating the need for rabies immunoglobulin administration after exposure and simplifying the PEP vaccine regimen. Compared to intradermal administration of the current lyophilized rabies vaccine via injection, a rabies MAP presentation may also offer similar or greater levels of dose-sparing, which would minimize product costs compared to intramuscular administration.

### 3.8 Yellow fever

Although current yellow fever vaccines are highly effective, safe, and affordable [39], ongoing supply constraints have made it challenging to meet demand, particularly during outbreaks [40]. MAPs have several potential attributes that could make them particularly beneficial for use in campaigns and outbreak settings, which could help improve access. For instance, MAPs could enable dose-sparing to ease supply constraints, eliminate reconstitution, and improve thermostability. In addition, in countries where yellow fever vaccine is included in the routine immunization schedule, co-administering MAP presentations for both yellow fever and MCV would simplify logistics compared to co-administering MAP and injectable vaccine presentations.

### 3.9 Influenza virus (seasonal and pandemic)

The potential MAP benefits related to increased thermostability, improved ease of use, and enhanced immunogenicity could be particularly beneficial for annual seasonal influenza vaccination as well as administration during outbreaks and/or pandemic response. Moreover, influenza vaccines are administered to a wide target population, including pregnant individuals, who could benefit from alternative delivery scenarios and administration in a community setting. Although the current seasonal influenza vaccine market in LMICs is small, there is a large global market with a need for annual administration. Global demand has also been increasing since the onset of the COVID-19 pandemic. Furthermore, if an influenza strain with pandemic potential were to be identified, having an approved seasonal influenza MAP could facilitate developing a pandemic influenza MAP by simplifying the product development pathway and expediting the time to market. Without an already approved seasonal influenza vaccine MAP, it is unlikely that a MAP presentation could be developed rapidly for use during an outbreak and/or pandemic.

### 3.10 SARS-CoV-2

Although the future market for annual COVID-19 vaccines in LMICs is uncertain, MAPs offer several attributes that would be useful during COVID-19 outbreak response, including improved thermostability, increased ease of use, self-administration, avoidance of reconstitution/dilution, dose-sparing, and enhanced immunogenicity. A MAP could also facilitate delivery to target populations outside of infants, such as adolescents and adults.

### 3.11 Group B streptococcus

GBS is a leading cause of neonatal/infant sepsis and meningitis, which is an important driver of antibiotic use and may contribute to antimicrobial resistance [41]. Maternal infection can also result in maternal sepsis, stillbirth, and preterm birth [41]. MAPs could be used as a delivery platform for vaccines administered during pregnancy, including pipeline GBS vaccines [42,43]. In addition, their increased ease of use could facilitate administration by lesser-trained personnel in antenatal care facilities as well as community settings. Moreover, as additional vaccines are recommended for use during pregnancy, a MAP presentation could reduce the number of injections required during pregnancy, which may increase acceptability and uptake of maternal vaccines more broadly. Although the greatest disease burden is in LMICs, GBS is also a public health concern in HICs, which could enable a dual market.

### 3.12 Neisseria meningitidis A,C,W,Y,(X)

Meningococcal disease burden remains significant globally despite major successes such as the elimination of serogroup A disease following the introduction of an affordable monovalent A conjugate vaccine (MenAfriVac®) in Africa [44]. However, other serogroups still cause cases and outbreaks in Africa and other regions where broadly protective and affordable meningococcal vaccines have not yet been introduced into routine immunization programs. To address this protection gap, an affordable injectable pentavalent ACWYX meningococcal conjugate vaccine (Men5CV) was WHO prequalified in 2023. However, access to Men5CV campaigns (preventive or reactive) could be expanded if a MAP presentation were available that increased ease of use, reduced cold chain requirements, and eliminated sharps waste. A MAP may also enable dose-sparing, which could reduce costs. Although the specific serogroups included in meningococcal vaccines vary by geography, there is a commercially attractive market for a quadrivalent ACWY meningococcal conjugate vaccine MAP in HICs, which could enable development of a pentavalent Men5CV MAP for LMICs. Combination meningococcal vaccines that protect against serogroup B could also be of interest to both LMIC and HIC markets, but the additional antigen would further increase the technical complexity of meningococcal MAP development.

### 3.13 Salmonella typhi

There is a high burden of typhoid fever in many parts of the world, and the prevalence of antimicrobial-resistant strains of *S. typhi* is increasing. Although effective vaccines against typhoid are available and injectable typhoid conjugate vaccines (TCVs) are being rolled out in routine immunization programs in some endemic countries, equitable vaccine coverage could be expanded if a MAP presentation were available. For instance, MAP attributes related to increased ease of use, delivery by lesser-trained personnel, improved safety, and elimination of sharps waste could make TCV MAPs particularly useful in campaign settings (both outbreak response and catch-up campaigns), expand access to hard-to-reach populations, and facilitate vaccine administration in camps for displaced people. MAPs may also facilitate stockpiling of TCVs [45].

### 3.14 Streptococcus pneumoniae

*Streptococcus pneumoniae* is the leading cause of lower respiratory tract infection morbidity and mortality worldwide, and one of the most common causes of meningitis in children less than 5 years of age [46,47]. Pneumococcal conjugate vaccines (PCVs) are widely used in immunization programs globally to protect against pneumococcal disease. PCVs also have one of the highest revenues of any vaccine in HICs, increasing the commercial viability of developing a PCV MAP with dual-market potential. Although pneumococcal vaccines are a critical tool for reducing child mortality, PCV is typically administered to infants during routine immunization visits alongside other injectable vaccines, which could reduce the added value of a PCV MAP compared to other vaccine MAPs that could be administered as standalone products outside of facility settings. Examples of nonroutine settings where PCV MAPs could be particularly beneficial include multi-age cohort campaigns and humanitarian settings. If a MAP presentation were to enable dose-sparing, this could also help to reduce the cost per dose and potentially make PCVs more affordable.

## 4 Discussion

Our prioritization process identified a list of high-priority vaccine targets for MAPs that could be of programmatic value to LMICs. Starting from a reference list of 91 vaccine targets, we applied a series of filters to generate an interim list of 21 vaccine targets, which was further narrowed down to a final VIPS priority list of 11 vaccines categorized into two priority groups.

At the time of this analysis, MAP preclinical or early-stage clinical studies had been initiated for 62 % (*n* = 13/21) and 64 % (*n* = 7/11) of the vaccines on the interim and final VIPS priority lists, respectively. This suggests alignment between the ongoing work in the MAP field and the VIPS Alliance's process for selecting potential MAP targets. These lists also represent a range of different vaccine types and platforms, highlighting that MAPs have the potential to be broadly applicable to a variety of vaccines. Inclusion of several vaccines against pathogens that have epidemic and/or pandemic potential, including SARS-CoV-2 and influenza, reinforces the role that MAPs could have in outbreak response. By acting on these priorities, stakeholders can ensure that MAP research and development investments better respond to global health needs. Integrating MAPs into broader health systems strengthening initiatives could also further improve health outcomes by addressing systemic issues in addition to vaccine-specific challenges.

Since the prioritization process described herein focuses on MAPs for LMIC contexts, other stakeholders may weigh the parameters differently and/or use different selection criteria. For instance, MAP developers may put more weight on the potential global market size or probability of technical and regulatory success to de-risk their product development efforts, and place less emphasis on the potential programmatic impact of a MAP application. Considering the status of vaccine development, we selected vaccines that were in phase 3 clinical development and beyond, except for outbreak pathogens, which reduces the risk of product failure when formulating a vaccine in a MAP presentation and ensures that efficacy and safety data have already been generated for the vaccine in its current presentation (e.g., liquid injectable). However, MAP developers may prefer to work with vaccines in preclinical or early-stage clinical development, which may be easier to access, offer more favorable commercial terms, and provide more options with regard to vaccine candidates and vaccine manufacturers compared to vaccines in late-stage clinical development. Since selection criteria are stakeholder- and context-specific, vaccine targets that are not on the final priority list should not be regarded as being de-prioritized or as nonviable candidates for MAP delivery. Although stakeholders may prioritize vaccine MAPs differently, our approach provides valuable insights into how to assess trade-offs relevant to the value proposition of vaccine MAPs. Moreover, our approach emphasizes the potential MAP use cases including alternative delivery scenarios since MAPs are expected to offer significant programmatic benefit when used outside of facility-based infant immunization visits.

Prioritization of vaccines for use with MAPs involves evaluating and balancing many different parameters. In producing the interim list, we aimed to use more objective, quantitative measures based on filtering criteria in our Excel database. However, as some data are not available for all vaccine targets, we used proxy measures to account for missing data. For instance, we used whether a vaccine is needed to combat antimicrobial resistance, if an elimination agenda has been set, and epidemic/pandemic potential as proxies for disease burden. In addition, many of the selection criteria are binary, which does not fully capture the possible values of each parameter. Moreover, the same level of detail was not available for all vaccines for each parameter. In the second stage of the prioritization process, we incorporated qualitative data based on stakeholder feedback to generate the final priority list. Although this enabled us to obtain critical insights into the nuances of prioritizing vaccine applications for MAPs, expert opinions are inherently subjective. To address this limitation, we selected diverse, independent experts and validated our findings through a public consultation process, which increased the breadth of feedback captured. By combining both quantitative and qualitative data inputs into our prioritization process, we were able to gain a more comprehensive understanding of high-priority vaccine applications for MAPs while mitigating the limitations of both methodological approaches. Furthermore, our results represent a snapshot in time, incorporating publicly available information as of February 2022, and do not fully capture the dynamic nature of product development. As new data are generated, our results could be updated to incorporate progress in the MAP field to provide guidance on which vaccine MAP candidates should be prioritized next.

## 5 Conclusions

This prioritization process highlighted the most advanced vaccine MAP candidates currently in early-stage clinical development, including MR, influenza, SARS-CoV-2, and hepatitis B, as well as new vaccine MAP concepts that could be high-impact targets for future MAP research and investments. VIPS partners are now working to address additional evidence gaps to accelerate MAP introduction timelines for LMIC markets, including generating clinical data, clarifying the regulatory and policy pathway, and conducting demand forecasts. Moreover, total cost of delivery and cost-effectiveness analyses are being conducted to ensure affordable access to vaccine MAPs in LMICs. To facilitate successful introduction and sustainable MAP use in LMICs, future studies should also focus on in-country evaluations and implementation research. Since the commercial viability of MAPs can be strengthened by advancing a balanced portfolio of MAP products, in terms of risk and the resources needed to bring MAP products to market, these findings could inform MAP developers, vaccine manufacturers, donors and financiers, as well as global health partners when deciding which global health MAP applications to advance within their own portfolios.

## Funding

PATH's contributions to this work were supported by the Gates Foundation, Seattle, Washington, USA [INV-041275; INV-062084]. Co-authors from other organizations received no specific funding for this work.

## Disclaimer

The authors alone are responsible for the views expressed in the article and they do not necessarily represent the views, decisions, or policies of the funders and the institutions with which they are affiliated.

## CRediT authorship contribution statement

**Collrane Frivold:** Writing – review & editing, Writing – original draft, Visualization, Methodology, Formal analysis, Data curation, Conceptualization. **Birgitte Giersing:** Writing – review & editing, Methodology, Conceptualization. **Jean-Pierre Amorij:** Writing – review & editing, Methodology, Conceptualization. **Mercy Mvundura:** Writing – review & editing, Conceptualization. **Mateusz Hasso-Agopsowicz:** Writing – review & editing, Conceptualization. **Jessica Joyce Mistilis:** Writing – review & editing, Conceptualization. **Kristen Earle:** Writing – review & editing, Conceptualization. **Courtney Jarrahian:** Writing – review & editing, Methodology, Funding acquisition, Conceptualization. **Marion Menozzi-Arnaud:** Writing – review & editing, Supervision, Methodology, Conceptualization. **Tiziana Scarna:** Writing – review & editing, Writing – original draft, Visualization, Methodology, Formal analysis, Data curation, Conceptualization.

## Declaration of competing interest

The authors declare that they have no known competing financial interests or personal relationships that could have appeared to influence the work reported in this paper.

## Data Availability

Data will be made available on request.

## Appendix A Supplementary data

Supplementary material 1 Image 1

Supplementary material 2: Summary of potential drivers for and key risks of developing vaccine MAP applications on the VIPS priority list. Image 2
