# Supplementary material for: Identification of potential vaccines for use with microarray patches in low- and middle-income countries: An assessment from the Vaccine Innovation Prioritisation Strategy Alliance
Source: Vaccine. 2025 May 10;55:None. doi: 10.1016/j.vaccine.2025.126996 (PMC12094179; doi:10.1016/j.vaccine.2025.126996)
Supplement: Supplementary file 1 — Supplementary material 1 [file mmc1.docx]

**Supplemental Data 1**

**Supplement to:** Identification of potential vaccines for use with microarray patches in low- and middle-income countries: An assessment from the Vaccine Innovation Prioritisation Strategy Alliance

**Supplemental Table 1. Reference list of vaccine targets (n=91).***

| ID | Target |
| --- | --- |
| 1 | Acinetobacter |
| 2 | Ascaris lumbricoides |
| 3 | Bacillus anthracis |
| 4 | Bordetella pertussis (aP and wP) |
| 5 | Borrelia burgdorferi |
| 6 | Campylobacter jejuni |
| 7 | Chikungunya virus |
| 8 | Chlamydia trachomatis |
| 9 | Clostridium difficile |
| 10 | Crimean-Congo hemorrhagic fever virus |
| 11 | Cytomegalovirus |
| 12 | Dengue virus |
| 13 | Diphtheria-tetanus (dT, low dose) |
| 14 | Diphtheria-tetanus-pertussis (DTaP and DTwP) |
| 15 | Diphtheria-tetanus-pertussis-polio (DTaP-IPV) |
| 16 | Diphtheria-tetanus-pertussis-polio-Haemophilus influenzae (DTaP-IPV-Hib) |
| 17 | Diphtheria-tetanus-pertussis-Haemophilus influenzae (DTwP-Hib) |
| 18 | Diphtheria-tetanus-pertussis-hepatitis B (DTwP-HepB) |
| 19 | Diphtheria-tetanus-pertussis-hepatitis B-Haemophilus influenzae-polio, hexavalent (DTaP-HepB-Hib-IPV) |
| 20 | Diphtheria-tetanus-pertussis-hepatitis B-Haemophilus influenzae, pentavalent (DTwP-HepB-Hib) |
| 21 | Dracunculus medinensis |
| 22 | Ebola virus |
| 23 | Echinococcus granulosis |
| 24 | Enterotoxigenic Escherichia coli (ETEC) |
| 25 | Enterovirus A71 |
| 26 | Epstein Barr virus (EBV) |
| 27 | Extraintestinal Escherichia coli (ExPEC) |
| 28 | Group A streptococcus (GAS; Streptococcus pyogenes) |
| 29 | Group B streptococcus (GBS; Streptococcus agalactiae) |
| 30 | Haemophilus influenzae type b (Hib) |
| 31 | Helicobacter pylori |
| 32 | Hepatitis A virus |
| 33 | Hepatitis B virus |
| 34 | Hepatitis C virus |
| 35 | Hepatitis E virus |
| 36 | Herpes simplex virus type 2 |
| 37 | Human herpesvirus 6 |
| 38 | Human herpesvirus 7 |
| 39 | Human herpesvirus 8 |
| 40 | Human hookworm |
| 41 | Human immunodeficiency virus 1 (HIV-1) |
| 42 | Human papillomavirus (HPV) |
| 43 | Influenza virus (pandemic) |
| 44 | Influenza virus, seasonal + universal |
| 45 | Japanese encephalitis virus (JE) |
| 46 | Klebsiella |
| 47 | Lassa fever virus |
| 48 | Leishmania donovani |
| 49 | Marburg virus |
| 50 | Measles |
| 51 | Measles-mumps-rubella-varicella viruses (MMRV) |
| 52 | Measles-mumps-rubella viruses (MMR) |
| 53 | Measles-rubella viruses (MR) |
| 54 | MERS coronavirus (MERS-CoV) |
| 55 | Mycobacterium leprae |
| 56 | Mycobacterium tuberculosis (bacillus Calmette-Guérin and next-generation) |
| 57 | Neisseria gonorrhoeae |
| 58 | Neisseria meningitidis (polyvalent and meningitis A) |
| 59 | Nipah virus and henipaviral diseases |
| 60 | Non-typhoidal salmonella (NTS) |
| 61 | Norovirus |
| 62 | Onchcocerca volvulus |
| 63 | Pathogen X |
| 64 | Plasmodium falciparum/vivax |
| 65 | Poliovirus, inactivated (IPV) and OPV bi- and monovalent |
| 66 | Pseudomonas aeruginosa |
| 67 | Rabies virus |
| 68 | Respiratory syncytial virus (RSV) |
| 69 | Rift Valley fever virus (RVF) |
| 70 | Rotavirus (next-generation and oral, live attenuated) |
| 71 | Rubella virus |
| 72 | Salmonella paratyphi |
| 73 | Salmonella Typhi |
| 74 | SARS-CoV-1 virus |
| 75 | SARS-CoV-2 virus |
| 76 | Schistosomiasis |
| 77 | Shigella (flexneri/sonnei) |
| 78 | Staphylococcus aureus |
| 79 | Streptococcus pneumoniae |
| 80 | Taenia solium |
| 81 | Tetanus (Clostridium tetani) |
| 82 | Tick-borne encephalitis virus |
| 83 | Treponema pallidum |
| 84 | Trichomonas vaginalis |
| 85 | Trypanosoma cruzi |
| 86 | Trypanosoma brucei |
| 87 | Varicella zoster virus (VZV), varicella/zoster |
| 88 | Vibrio cholerae (next-generation and OCV) |
| 89 | Yellow fever virus (YF) |
| 90 | Yersinia pestis |
| 91 | Zika virus |

* Some vaccines and vaccine targets that are on the US Department of Defense list of potential biological warfare threats were not included, if it was judged that the main risk of infection was from biological warfare rather than from natural reservoirs of the pathogen. Vaccines not included: Brucella species (brucellosis), Clostridium perfringens, Coxiella burnetii (Q fever), Francisella turalensis, Variola major (smallpox), Botulinum toxins A–G, Staphylococcal enterotoxins A and B, Ricin toxin.

**Supplemental Table 2. External advisory group members.**

| **Name** | **Affiliation (as of 2022)** |
| --- | --- |
| Jon Abramson | Professor Wake Forest School of Medicine  Form*er WHO Strategic Advisory Group of Experts on Immunization (SAGE) Member* |
| Moredreck Chibi | Local production of pharmaceuticals World Health Organization (WHO) |
| Amos Chweya | Director, Regional Immunization Advisor  InSupply Health, JSI |
| Alejandro Cravioto | Professor Universidad Nacional Autónoma de México  *Former WHO SAGE Member*  *WHO Product Development for Vaccines Advisory Committee (PDVAC) Member* |
| Renske Hesselink | Senior CMC Scientist  Coalition for Epidemic Preparedness Innovations |
| Jaleela Jawad | Minister of Health, Kingdom of Bahrain  *WHO SAGE Member*  *Former WHO PDVAC Member*  *Former Head of Immunization Group and EPI Manager Ministry of Health* |
| Gagandeep (Cherry) Kang | Professor  Christian Medical College Vellore  *India Chair, Regional Immunization Technical Advisory Group*  *Former WHO PDVAC Member* |
| David Kaslow | Chief Scientific Officer for Essential Medicines PATH  *Former WHO PDVAC Member* |
| Jerome Kim | Director General International Vaccine Institute  *Former WHO PDVAC Member* |
| Yanfeng Lim | Vaccines Market Team  Clinton Health Access Initiative |
| Chris Morgan | Senior Technical Advisor, Immunization Jhpiego |
| Nicaise Ndembi | Senior Science Advisor and Professor  Africa Centres for Disease Control and Prevention |
| David Robinson | Deputy Director, Vaccine Development & Surveillance  Gates Foundation |

**Supplemental Table 3. Evaluation of the expected complexity of the microarray patch regulatory pathway to inform the VIPS final priority list.**

**Low complexity:** Surrogate of efficacy identified

**Medium complexity:** Correlate of protection or immunological endpoint identified

**High complexity:** No correlate of protection nor immunological endpoint identified

|  | **Potential vaccine targets for use with MAPs** | **Expected complexity of the regulatory pathway** | |
| --- | --- | --- | --- |
| **Legacy**  High volumes of vaccines available with low unit price | **Hepatitis B virus** |  | **Surrogate of efficacy identified.** |
|  | **Measles and rubella viruses** |  | **Surrogate of efficacy identified.** |
|  | **Measles, mumps, and rubella viruses** |  | It is **likely that approval will be possible based on non-inferiority of immune responses** as done with other development efforts on novel delivery systems for MCVs. |
|  | **Rabies virus** |  | **Surrogate of efficacy identified.** |
|  | ***Salmonella* Typhi** |  | - Licensure of a TCV MAP should be possible **based on non-inferior immunogenicity** compared with an approved injected TCV. - **Data suggest that anti-Vi serum IgA is a correlate of protection** against infection and anti-Vi serum IgG is associated with protection against severe disease. |
|  | **Yellow fever** |  | **Neutralizing antibodies are regarded as a valid correlate of protection.** |
|  |  |  |  |
| **Evolving**  Not commoditized/higher-priced vaccines, or vaccines still in development | **Group B streptococcus (*Streptococcus agalactiae*)** |  | - **Correlates of protection are likely to be required** for initial licensure of a GBS vaccine because a pivotal vaccine efficacy trial would require a very large sample size. - **Antibody-mediated risk reduction estimates have been reported** from different studies for anti-capsular antibodies against the most common serotypes of GBS. - **The concentration of antibodies required for protection has not been defined.** - For vaccines indicated for use in pregnancy, like the GBS vaccine, pregnant individuals should be prioritized for inclusion in early-phase clinical development to prevent delays in approval for use in pregnancy. |
|  | **Human papillomavirus** |  | A **correlate of protection for HPV vaccines has not been defined**. Demonstration of **non-inferiority of immune responses is expected to be an acceptable endpoint** for regulatory approvals. |
|  | ***Neisseria meningitidis* A,C,W,Y** |  | **Correlates of protection have been defined** for meningitis A,C,W,Y,X. |
|  | ***Neisseria meningitidis* A,C,W,Y,X** |  |  |
|  | ***Streptococcus pneumoniae*** |  | **Surrogate of efficacy identified.** |
|  |  |  |  |
| **Outbreak**  Vaccine targets with unpredictable demand driven by outbreaks | **Influenza virus (pandemic and seasonal)** |  | The **correlate of protection for pandemic flu vaccines is assumed to be the same as for seasonal flu vaccines** (surrogate of efficacy). |
|  | **SARS-CoV-2** |  | **Data suggest** serological response to vaccination (measured with Anti-Spike IgG) could be a valid **surrogate of efficacy**. |

Abbreviations: GBS, Group B streptococcus; HPV, human papillomavirus; IgA, immunoglobulin A; IgG, immunoglobulin G; MAP, microarray patch; MCV, measles conjugate vaccine; SARS-CoV-2, severe acute respiratory syndrome coronavirus 2; TCV, typhoid conjugate vaccine.

**Supplemental Table 4. Evaluation of potential programmatic impact to inform the VIPS final priority list.**

**
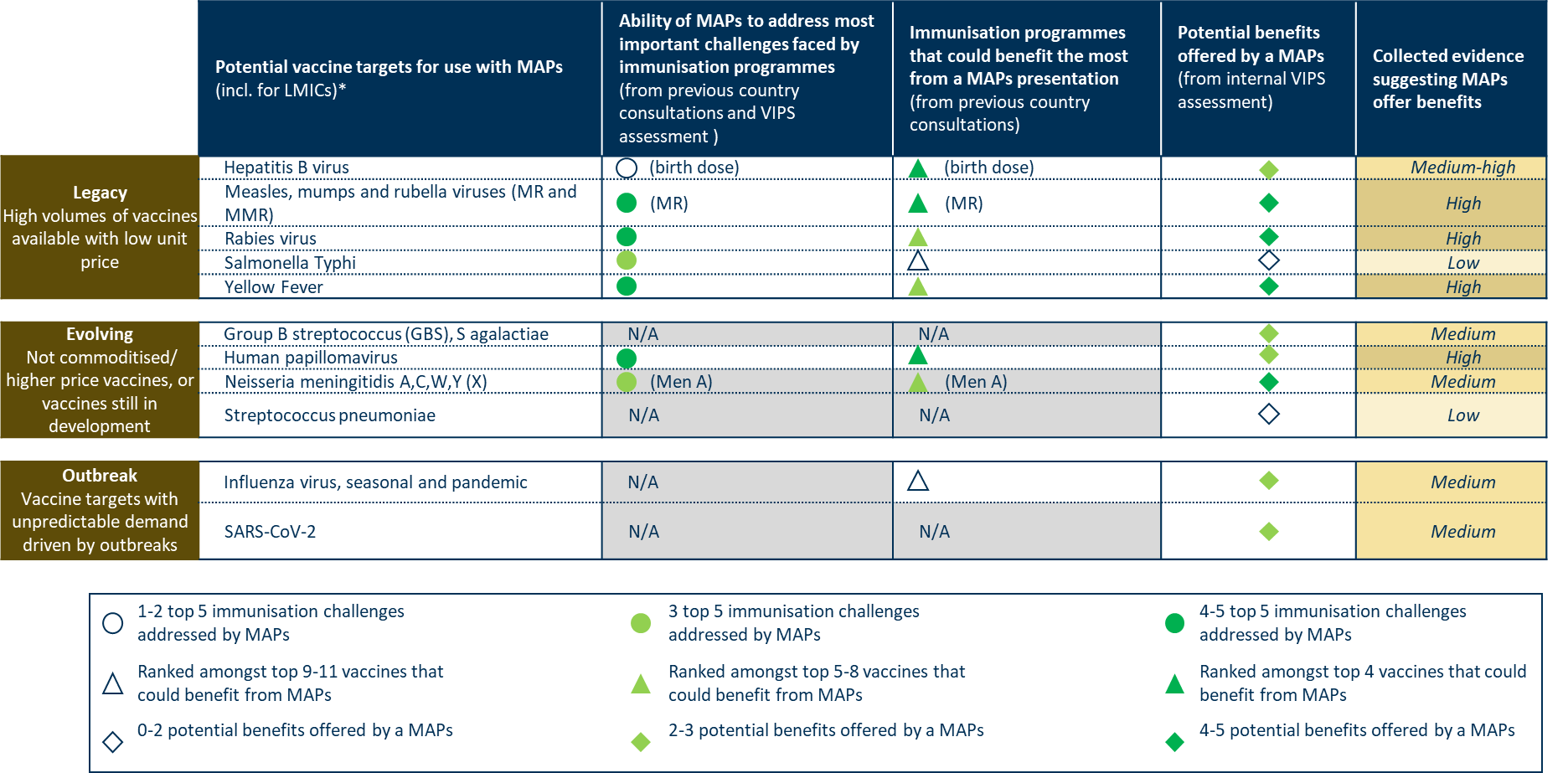
**

Abbreviations: LMICs, low- and middle-income countries; MAP, microarray patch; N/A, not applicable; SARS-CoV-2, severe acute respiratory syndrome coronavirus 2; VIPS, Vaccine Innovation Prioritisation Strategy.

**Supplemental Table 5. Evaluation of financial sustainability/funders’ interest to inform the VIPS final priority list.**

|  | **Potential vaccine targets for use with MAPs** (including for LMICs) | **Potential dual market (in HICs and LMICs)** | **Known funder; vaccine manufacturer or MAP developer interested in vaccine MAP** | **Vaccine MAP development status** | **Financial sustainability/ funders’ interest** |
| --- | --- | --- | --- | --- | --- |
| **Legacy**  High volumes of vaccines at low unit prices available | Hepatitis B virus | **Yes** | **Yes** | Clinical (adults) | *High* |
|  | Measles, mumps, and rubella viruses | **No** (MR)  **Yes** (MMR) | **Yes** (MR)  **No** (MMR) | Clinical (MR) | *Medium* |
|  | Rabies virus | **Maybe** (travelers and  high-risk occupations) | **Yes** | Preclinical | *Medium-high* |
|  | *Salmonella* Typhi | **Maybe** (travelers  and military) | **Yes** | Concept* | *Medium-high* |
|  | Yellow fever | **Maybe** (travelers) | **No** | Concept | *Medium-low* |
|  |  |  |  |  |  |
| **Evolving**  Not commoditized/ higher-priced vaccines, or vaccines still in development | Group B streptococcus (*Streptococcus agalactiae*) | **Yes** | **No** | Concept | *Medium* |
|  | Human papillomavirus | **Yes** | **Yes** | Preclinical | *High* |
|  | *Neisseria meningitidis* A,C,W,Y,(X) | **Maybe** (serotype X is less of a problem in HICs) | **No** | Concept | *Medium-low* |
|  | *Streptococcus pneumoniae* | **Yes** | **No** | Preclinical | *Medium* |
|  |  |  |  |  |  |
| **Outbreak**  Vaccine targets with unpredictable demand driven by outbreaks | Influenza virus (pandemic) | **Yes** | **Yes** | Clinical (seasonal) | *High* |
|  | SARS-CoV-2 | **Yes** | **Yes** | Clinical | *High* |

* Typhoid conjugate vaccine MAP preclinical work was initiated after February 2022.

Abbreviations: HIC, high-income country; LMICs, low- and middle-income countries; MAP, microarray patch; MMR, measles-mumps-rubella; MR, measles-rubella; SARS-CoV-2, severe acute respiratory syndrome coronavirus 2.

**Supplemental Table 6. Potential benefits offered by the microarray patch presentation to inform scoring of potential programmatic impact.**

| **Vaccine target** | **Potential benefits offered by a vaccine microarray patch** | | | | | | |
| --- | --- | --- | --- | --- | --- | --- | --- |
|  | **Dose-sparing/ immunogenicity** | **Thermostability** | **Missed opportunities** | **Ease of use/ self-administration** | **Acceptability** | **Avoid reconstitution** | **Count** |
| Group B streptococcus (*Streptococcus agalactiae*) | Yes | Yes | No | Yes | Possibly | No | 3 |
| Hepatitis B virus | Yes | To be determined | Yes | Yes | To be determined | No | 3 |
| Human papillomavirus | Yes | To be determined | No | Yes | Yes | No | 3 |
| Influenza virus (seasonal/pandemic) | Yes | Yes | No | Yes | No | To be determined | 3 |
| Measles-rubella viruses/measles-mumps-rubella viruses | Possibly | Yes | Yes | Yes | Possibly | Yes | 4 |
| *Neisseria meningitidis* A,C,W,Y,(X) | Yes | Yes | No | Yes | No | Yes | 4 |
| Rabies virus | Yes | Yes | Yes | Yes | Possibly | Yes | 5 |
| *Salmonella* Typhi | Yes | Possibly | No | Yes | Possibly | No | 2 |
| SARS-CoV-2 virus | Yes | Yes | Possibly | Yes | No | No | 3 |
| *Streptococcus pneumoniae* | Yes | Possibly | No | Yes | Possibly | No | 2 |
| Yellow fever | Yes | Yes | Yes | Yes | Possibly | Yes | 5 |

Abbreviation: SARS-CoV-2, severe acute respiratory syndrome coronavirus 2.
