# Supplementary material for: Identification of potential vaccines for use with microarray patches in low- and middle-income countries: An assessment from the Vaccine Innovation Prioritisation Strategy Alliance
Source: Vaccine. 2025 May 10;55:None. doi: 10.1016/j.vaccine.2025.126996 (PMC12094179; doi:10.1016/j.vaccine.2025.126996)
Supplement: Supplementary file 2 — Supplementary material 2: Summary of potential drivers for and key risks of developing vaccine MAP applications on the VIPS priority list. [file mmc2.pdf]

VIPS VACCINE INNOVATION PRIORITISATION STRATEGY

Identification of priority vaccines for use with microarray patches (MAPs): Technical Notes

Supplemental Data 2

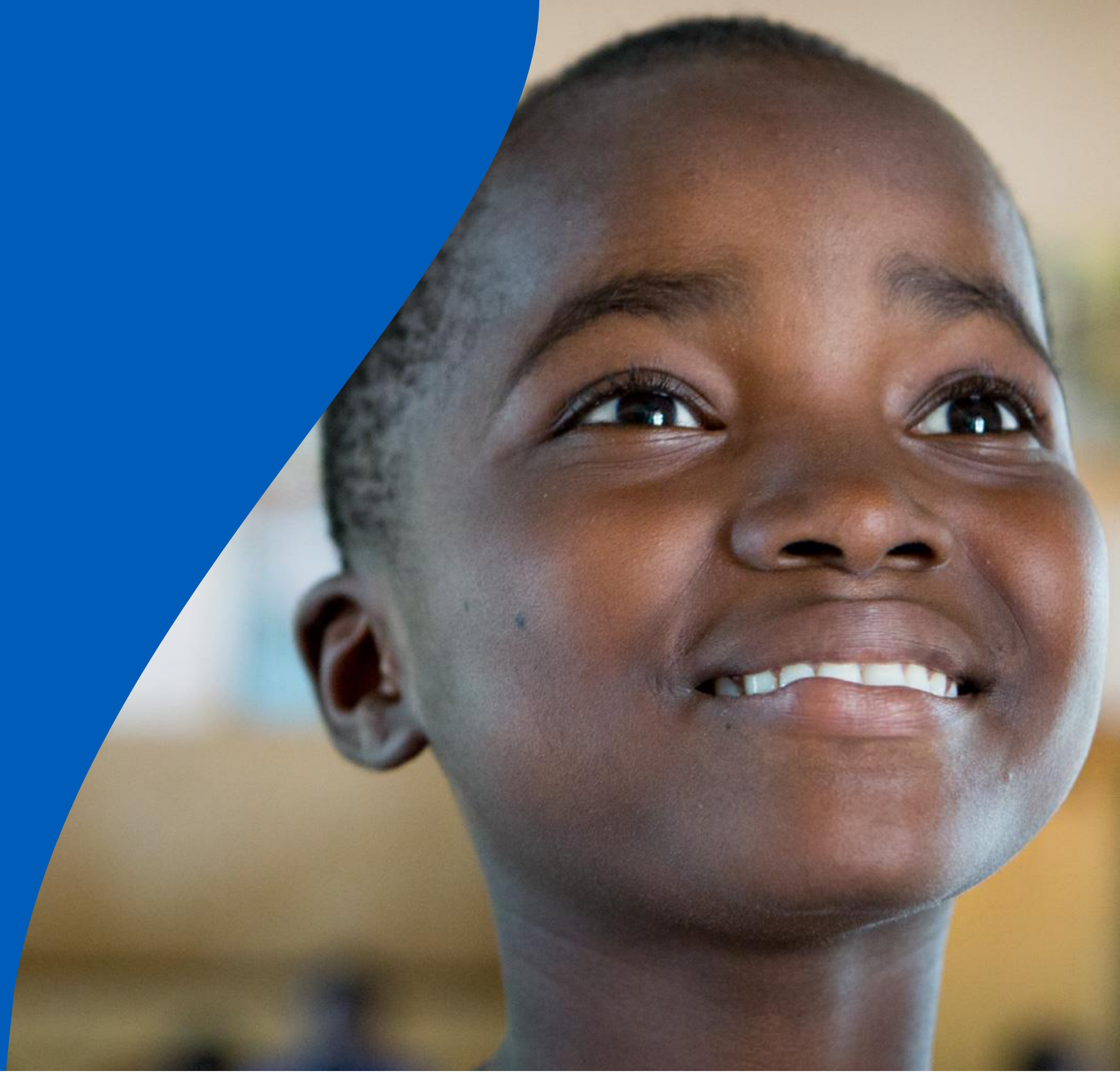

Gavi The Vaccine Alliance | World Health Organization | Bill & Melinda Gates foundation | PATH | unicef

Priority 1 group

Hepatitis B vaccine-MAPs: potential drivers for and key risks facing prioritisation for use with vaccine-MAPs

VIPS VACCINE INNOVATION PRIORITISATION STRATEGY

Potential drivers for developing Hep B vaccine-MAP

**Market Distribution**	In the USA at least, **the recommendation for adult use of HepB vaccines is increasing.** The results of a **recent cost-effectiveness** analysis suggest **that even at higher procurement costs** (e.g., US$1.65-5.00 per dose) **MAPs** are likely to provide a **cost-saving or cost-effective mechanism to increase hepatitis B birth dose coverage in LMICs.**
**Specific global health priority**	In 2016, the World Health Assembly endorsed a World Health Organization (WHO) **strategy to eliminate Hepatitis B (HepB) virus as a public health threat by 2030.**
**Potential programmatic impact offered by a vaccine MAP**	• **Thermostability:** A thermostable HepB MAP **might improve access to HepB birth dose (BD)** particularly for home-births (timely HepB birth dose coverage is low).<br>• **Ease of use: Access to HepB-BD vaccine** might be **improved** by a MAP that could be administered by **untrained or minimally trained individuals,** for home births.<br>• **Acceptability**: it is **possible that a HepB MAP might be more acceptable** than an injectable vaccine **to parents of infants at birth.**<br>• **Enhanced immunogenicity**. A **HepB MAP might be more immunogenic for some populations** (of all ages) that are known to be hard to immunise with existing formulations.

Key risks facing development of Hep B vaccine-MAP

**Market Distribution**	The value of developing a MAP for a very inexpensive widely used vaccine for LMICs is **uncertain**. The '**newer' vaccines that might be used in adults in HICs** such as the USA **might be too expensive.**
**Expected complexity of the regulatory pathway**	• HepB vaccines are adjuvanted. Preliminary clinical data suggest that it's feasible for formulate an **unadjuvanted HepB MAPs** that a MAP can induce a protective immune response, but the antibody titers may be lower compared to with the adjuvanted injectable formulation.<br>• There are **no data to date on the use of any vaccine MAPs in new-borns** (only adults). Their performance in the skin of new-borns might be different to in adults, adolescents or infants.

Measles and measles rubella viruses: potential drivers for and key risks facing prioritisation for use with vaccine-MAPs

VACCINE INNOVATION PRIORITISATION STRATEGY

Potential drivers for developing M and MR vaccine-MAPs

Known global health organisation interest	There is **considerable interest in MR MAPs** from a range of different stakeholders including **MAP developers (MDs), vaccine manufacturers (VMs) and global health organisations and funders,** including the VIPs Alliance as additional tools are needed to reach coverage/elimination goals.
Expected complexity of the regulatory pathway	**Two MR MAP products are currently in phase 1 trials and results are expected in 2022.** Therefore, **more risk has been removed from the development of MR MAPs than for many other vaccine MAPs.**
Potential programmatic impact offered by a vaccine MAP	Many of the **potential benefits offered by MAPs** should be **relevant to MR, including: thermostability, ease of use, avoidance of reconstitution and reduction of missed opportunities.**

Key risks facing development of M and MR vaccine-MAPs

Market Distribution	**Currently, there isn't a high-income country (HIC) market for MR MAPs.** In addition, **existing MR vaccines are relatively inexpensive.** Therefore, MR MAPs might not be commercially appealing to VMs or MDs. In addition, **MR MAPs are likely to have a higher price per dose than the current MR vaccines,** and purchasers might not be willing to pay the additional cost.
Expected complexity of the regulatory pathway	• The processes and **facilities needed for commercial scale manufacture have not been established.** These are on the critical path to product availability and WHO prequalification (PQ). However, funding to establish MR-MAP manufacturing facilities is not in place.<br>• **Developers' profiles:** MR MAPs are being developed by **two, relatively small MDs, and** the existing project relies on **MR vaccine from a single VM and donor funding.** Development of MR MAPs is at **risk of MDs failing to attract sufficient funding to operate, and/or lack of supply of MR vaccine.**

Human papillomavirus: potential drivers for and key risks facing prioritisation for use with vaccine-MAPs

VIPS VACCINE INNOVATION PRIORITISATION STRATEGY

Potential drivers for developing HPV-MAPs

**Market Distribution**	**Dual market: HPV vaccines are used in high income countries (HICs) and LMICs.** The LMIC market is believed to be growing. The **HIC market is very large.**
**Specific global health priority**	The **WHO has a Global Strategy to Accelerate the Elimination of Cervical Cancer** (2018), but **vaccine supply and access** are currently **barriers** and there are challenges to meet coverage targets for HPV.
**Population/ settings for potential use of vaccine MAP**	HPV vaccines are administered to **adolescents outside infant visits**, and in **multi-age cohort campaigns** (MACs).
**Potential programmatic impact offered by a vaccine MAP**	• There has been, and still is, **interest in intradermal (ID) delivery of human papillomavirus (HPV) vaccines to achieve dose-sparing.**<br>• MAP attributes such as **ease-of-use** and **thermostability** might be very valuable in facilitating delivery to target populations (adolescents outside infant visits).
**Commercial/ funders interest** (potential or existing)	There is **existing interest in HPV vaccine-MAPs** from various stakeholders.

Key risks facing development of HPV-MAPs

**Expected complexity of the regulatory pathway**	**Developing a MAP for a multivalent, currently adjuvanted vaccine is technically complex.** Data have been published on delivery of an unadjuvanted HPV MAP in non-human primates.
**Potential programmatic impact offered by a vaccine MAP**	The **potential transition to single-dose HPV vaccination schedules** (endorsed by SAGE in April 2022), coupled with improved supply of HPV vaccines in the future, could **weaken the value proposition for HPV vaccine MAPs.**

Rabies virus: potential drivers for and key risks facing prioritisation for use with vaccine-MAPs

VIPS VACCINE INNOVATION PRIORITISATION STRATEGY

Potential drivers for developing rabies vaccine-MAPs

Potential programmatic impact offered by a vaccine MAP

- **Setting for use.** A MAP presentation could facilitate administration of **post-exposure prophylaxis (PEP) at the community level by a lesser-trained health care worker, trained volunteer, or caregiver, or through self-administration.** This could reduce the number of clinic visits and improve compliance.
- **Dose-sparing. Rabies vaccines (RABV) are expensive. Intradermal (ID) administration is already used in some countries to reduce the cost of the vaccine.** RABV MAPs might offer similar or greater levels of dose-sparing.
- **Enhanced immunogenicity.** RABV MAPs might generate a protective immune response more quickly as has been seen with other vaccine MAPs. This would be an **advantage for PEP, where speed of the response after exposure is critical,** and also to PrEP of travellers.

Key risks facing development of rabies vaccine-MAPs

Market Distribution

- **The market for rabies vaccines is relatively small and fragmented with many manufacturers.** Future demand could be reduced by wider adoption of ID regimens.
- **Cost: rabies vaccines are already regarded as expensive and rabies vaccine-MAP products are likely to have a higher price per dose than existing presentations.** To be attractive to purchasers, RABV-MAPs may need to use significantly less antigen, and/or offer other benefits that offset the cost. This is even more a barrier to access as rabies vaccine is often times paid out-of-pocket.

Gavi The Vaccine Alliance · World Health Organization · BILL & MELINDA GATES foundation · PATH · unicef

Yellow fever virus: potential drivers for and key risks facing prioritisation for use with vaccine-MAPs

VIPS VACCINE INNOVATION PRIORITISATION STRATEGY

Potential drivers for developing yellow fever vaccine-MAPs

**Population/ settings for potential use of vaccine MAP**	**Use in outbreaks. MAPs have several potential attributes** that could make them particularly beneficial for use **in campaigns and outbreak settings.** These could help improve access to YF vaccines.
**Potential programmatic impact offered by a vaccine MAP**	**Dose-sparing**. If YF MAPs enabled **less vaccine to be used per dose, they could help to ease ongoing and future supply constraints.**

Key risks facing development of yellow fever vaccine-MAPs

**Market Distribution**	**Low commercial viability:** the YF vaccine market is not attractive for VMs. It is relatively small, with a low profit margin and demand is largely driven by outbreaks, which are unpredictable.
**Expected complexity of the regulatory pathway**	**Changing existing vaccines: YF vaccines are extremely effective. They are also associated with very rare, but severe adverse events**. Any **changes to the vaccine and/or delivery route, are likely to require lengthy clinical testing** to confirm that immunogenicity of vaccine MAPs is non-inferior and that the **risk of adverse events is not increased.**
**Potential programmatic impact offered by a vaccine MAP**	**Dose-sparing.** Fractional dosing is **already used with existing YF vaccines** without changing the route of delivery from subcutaneous (SC) or intramuscular (IM). If they are **to be attractive, YF MAPs would probably need to use even less vaccine per dose than the 1/5 fractional doses** that can be used currently, which may not be technically feasible.

Gavi The Vaccine Alliance | World Health Organization | Bill & Melinda Gates foundation | PATH | unicef

Seasonal/Pandemic Flu: potential drivers for and key risks facing prioritisation for use with vaccine-MAPs

VIPS VACCINE INNOVATION PRIORITISATION STRATEGY

Potential drivers for developing Seasonal/Pandemic Flu vaccine-MAPs

Specific global health priority

WHO recommends that all countries should consider implementing seasonal flu immunization programmes, but the vaccine is not widely available in LMICs. Avian strains of influenza have **pandemic potential** once human-to-human transmission is established.

Potential programmatic impact offered by a vaccine MAP

- MAPs have several **potential attributes** would be valuable for use in a **pandemic flu response.**
- MAPs could **improve access** to seasonal flu vaccine, particularly when administered outside of EPI visits to high-risk populations (children <5; pregnant people; elderly; individuals with comorbidities).

Market Distribution

Dual market: Seasonal flu vaccines are used in HICs. The LMIC market for seasonal flu is currently small, but a MAP presentation could increase uptake and **demand has been increasing in recent years**. MAP developers could potentially offer both seasonal and pandemic flu MAP products to expedite pandemic response efforts.

Expected complexity of the regulatory pathway

Seasonal flu MAPs are the **most advanced vaccine-MAPs** with multiple Phase 1 clinical studies completed.

Key risks facing development of Seasonal/Pandemic Flu vaccine-MAPs

Market Distribution

The **commercial opportunity or business model** for pandemic flu vaccines is **unclear** in LMICs.

Potential programmatic impact offered by a vaccine MAP

Avian **strains of influenza viruses such as H5N1 or H7N9 that could cause a pandemic are often poorly immunogenic**. Although a MAP can increase immunogenicity, it may not be able to overcome some of the barriers of current injectable vaccines. Adjuvants not compatible with MAPs may be required.

Gavi The Vaccine Alliance | World Health Organization | BILL & MELINDA GATES foundation | PATH | unicef

SARS-CoV-2: potential drivers for and key risks facing prioritisation for use with vaccine-MAPs

VIPS VACCINE INNOVATION PRIORITISATION STRATEGY

Potential drivers for developing SARS-CoV-2 vaccine-MAPs

**Potential programmatic impact offered by a vaccine MAP**	▪ **Improved thermostability.** Some **COVID-19 vaccines need to be stored and distributed at -20°C or -80°C,** which is particularly challenging for vaccines to control outbreaks.<br>▪ **Enhanced immunogenicity.** A more rapid onset of a protective immune response, and/or **dose-sparing to 'stretch' manufacturing capacity, would be beneficial.**<br>▪ **Ease of use.** Facilitating **administration by lesser trained personnel and/or self-administration** might **improve access to vaccines.**
**Population/ settings for potential use of vaccine MAP**	▪ **Demonstration of the compatibility (or not) of MAPs with a range of vaccine platforms**. This experience would be valuable and **support future rapid development of vaccines against Pathogen X.**
**Commercial/ funders interest** (potential or existing)	▪ **BARDA** is funding development of **COVID-19-MAPs by three MDs.** The Right Fund is supporting at least one MD to develop COVID-19-MAPs.

Key risks facing development of SARS-CoV-2 vaccine-MAPs

**Expected complexity of the regulatory pathway**	▪ **Some platforms will be technically challenging** to formulate for MAPs.<br>▪ Evolution of the COVID-19 pandemic means that clinical development and large-scale efficacy trials in particular, will be difficult.<br>▪ Choice and type of vaccines. MAP developers (MDs) and vaccine manufacturers (VMs) need to decide if they are developing **vaccines for primary immunisation or booster vaccinations. Different vaccines might be preferable for the different indications.**
**Market Distribution**	▪ **Commercial uncertainty.** Whether or not there is a long-term need for booster vaccines needs to be determined.<br>▪ Rapid, large-scale production of COVID-19 vaccine MAPs will be needed for a MAP product to add value to the pandemic response. Currently, the processes and **facilities required for larger, commercial-scale manufacture have not been developed for vaccine MAPs. These might not be in place in time to have an impact on the COVID-19 pandemic.**

Priority 2 group

Group B streptococcus: potential drivers for and key risks facing prioritisation for use with vaccine-MAPs

VIPS VACCINE INNOVATION PRIORITISATION STRATEGY

Potential drivers for developing Group B streptococcus vaccine-MAPs

**Market Distribution**	**Group B streptococcus (GBS) vaccine-MAPs could have markets in HICs as well as LMICs.** This might make them more appealing to vaccine manufacturers (VMs) and MAP developers (MDs) than other vaccine MAPs for LMICs.
**Population/ settings for potential use of vaccine MAP**	**MAPs could be used as a 'delivery platform' for vaccines administered during pregnancy**. Tetanus and influenza vaccines are also recommended for pregnant people, and maternal immunisation could benefit from MAP administration if delivered by a skilled birth attendant and/or midwife
**Potential programmatic impact offered by a vaccine MAP**	MAPs could offer benefits such as **ease-of-use that might be beneficial in ante-natal settings**. It might also be possible to develop next-generation MAPs delivering two or more maternal vaccines.

Key risks facing development of Group B streptococcus vaccine-MAPs

**Expected complexity of the regulatory pathway**	**Limited choice of GBS vaccine candidates**. There are no licensed GBS vaccines, and there is only one candidate GBS vaccine that is understood to have completed phase 2 proof of concept and is advancing to phase 3. There is therefore **still significant risk that this vaccine will not achieve success in phase 3 and be approved.**

Gavi The Vaccine Alliance · World Health Organization · Bill & Melinda Gates foundation · PATH · unicef

Meningitis viruses: potential drivers for and key risks facing prioritisation for use with vaccine-MAPs

VACCINE INNOVATION PRIORITISATION STRATEGY

Potential drivers for developing Meningitis vaccine-MAPs	
**Specific global health priority**	There is a **need to vaccinate against additional meningococcal serotypes, beyond group A** (MenAfriVac) in sub-Saharan Africa, particularly groups W and X.
**Potential programmatic impact offered by a vaccine MAP**	NmCV-5 (and possibly NmCV-4) **could be used in campaigns in LMICs;** the NmCV-5 candidate currently in phase 3 trials is **lyophilised**. A MAP may enable **dose sparing**, which could reduce the total cost of delivery per dose.
**Market Distribution**	There is potentially a **commercially attractive market for NmCV-4 MAPs in HICs.**
**Expected complexity of the regulatory pathway**	Data suggest that it should be **technically feasible to develop NmCV-4 and NmCV-5 MAPs.**

Key risks facing development of Meningitis vaccine-MAPs	
**Market Distribution**	There are likely to be **different preferences for serogroups for Men MAPs in HIC and LMIC markets**; HICs are likely to prefer vaccines that include serogroup B rather than group X.
**Expected complexity of the regulatory pathway**	Some **VMs are already developing vaccines that include Men A,C,W,Y and B for HIC markets**. These are likely to be more difficult to formulate for use with MAPs than NmCV-4 and -5.

Salmonella Typhi: potential drivers for and key risks facing prioritisation for use with vaccine-MAPs

VACCINE INNOVATION PRIORITISATION STRATEGY

Potential drivers for developing TCV-MAPs

Specific global health priority	There is a **high burden of typhoid fever** in many parts of the world and **increasing prevalence of antimicrobial resistant (AMR) strains of S. Typhi.**
Potential programmatic impact offered by a vaccine MAP	MAPs **could increase access to TCVs in low- and middle-income countries (LMICs),** especially in **campaigns (outbreak response and catch-up campaigns) and, possibly, in camps for displaced people.**
Commercial/ funders interest (potential or existing)	There is **interest from research and development (R&D) funders,** such as BMGF and Wellcome Trust, in typhoid prevention including in the accelerated introduction and use of TCVs. There is interest from at least one funder, VM, and MD in advancing a TCV MAP.
Expected complexity of the regulatory pathway	Development of **TCV MAPs is likely to be technically feasible and TCV MAPs could act as exemplars for** other, **more complex** polysaccharide-protein conjugate **vaccines** (PS-PCVs) such as meningitis and pneumococcal pneumonia.

Key risks facing development of TCV-MAPs

Market Distribution	The future demand for TCVs is **not well understood**; there could be **excess TCV supply** from multiple manufacturers around the time a TCV MAP could come to market.<br>It is possible, but not known, if there will be a market for TCV MAPs as travel vaccines and/or a private market in countries such as India.
Population/ settings for potential use of vaccine MAP	The **use case(s)** for TCV MAPs at the time of introduction are **unknown**.
Vaccine target information	A **combination TCV product with paratyphoid and/or non-typhoidal salmonella (NTS)** vaccines might be preferable.

Streptococcus pneumoniae: potential drivers for and key risks facing prioritisation for use with vaccine-MAPs

VIPS VACCINE INNOVATION PRIORITISATION STRATEGY

Potential drivers for developing PCV-MAPs

Market Distribution	Pneumococcal conjugate vaccines (PCVs) are **recommended for routine immunisation of infants in HICs, middle-income countries (MICs), as well as LMICs. PCVs have the highest revenues of any vaccine in HICs.** However, there is a trend for VMs to **develop higher valency PCVs for HICs** which are likely to be **less affordable for self-procuring LMICs and MICs**.
Potential programmatic impact offered by a vaccine MAP	**Dose-sparing.** If PCV MAPs were found to be dose-sparing, this **might help to reduce the cost per dose and make PCVs more affordable.**

Key risks facing development of PCV-MAPs

Expected complexity of the regulatory pathway	• All **PCVs are currently adjuvanted.** It is **not known whether the adjuvants used will be compatible with MAPs,** whether new adjuvants will be required, or whether unadvantaged PCV MAPs will be immunogenic.<br>• **PCVs are multi-valent, containing polysaccharide (PS) from seven to twenty different serotypes.** This will present **significant technical challenges for MDs,** particularly when confirming the dose of vaccine loaded, stability, and the dose of vaccine delivered.
Population/ settings for potential use of vaccine MAP	The **vast majority of PCVs are administered to infants as part of routine immunization schedules,** alongside DTP-containing vaccines. Although some of **MAPs' potential attributes would be beneficial in these settings, they might not provide as much value** as they would to other vaccines such as those used in outreach settings.

Gavi The Vaccine Alliance · World Health Organization · Bill & Melinda Gates foundation · PATH · unicef
